# A Docking-Guided Strategy Prioritizes Parishin B as a Potential Anti-Alzheimer’s Constituent of *Gastrodia elata* Blume

**DOI:** 10.3390/biology15201830

**Published:** 2026-10-13

**Authors:** Jia Wang, Mingming Rong, Jianlin Cui, Heng Guo, Jing Xu, Yuanqiang Guo

**Affiliations:** 1 Institute of Traditional Chinese Medicine, Tianjin University of Traditional Chinese Medicine, Tianjin 301617, China; 18830931563@163.com; 2 State Key Laboratory of Medicinal Chemical Biology, College of Pharmacy, and Tianjin Key Laboratory of Molecular Drug Research, Nankai University, Tianjin 300350, China; 18519232516@163.com (M.R.); victgyq@nankai.edu.cn (Y.G.); 3 School of Medicine, Nankai University, Tianjin 300071, China; 4 Department of Pharmacy, Wuhan No. 1 Hospital, Wuhan 430030, China

**Keywords:** *Gastrodia elata* Blume, Alzheimer’s disease, Parishin B, Phosphodiesterase 4B (PDE4B), molecular docking, neuroinflammation

## Abstract

Alzheimer’s disease (AD) is a progressive neurodegenerative disorder with limited treatment options. Phosphodiesterase 4B (PDE4B) is a key regulator of neuroinflammation and has been implicated in AD pathology. Using molecular docking against PDE4B as a screening anchor, we analyzed constituents from *G. elata* water extract and identified Parishin B as a top-ranked candidate. Parishin B directly inhibited recombinant PDE4B, reducing activity to 22.1% of control at 100 μM. In an Al^3+^-induced zebrafish AD model, both the crude extract and purified Parishin B significantly rescued behavioral deficits; Parishin B at 100 μM showed no statistical difference from the donepezil group, while the crude extract was effective at 30 μg/mL. Mechanistically, Parishin B protected SH-SY5Y neurons against H_2_O_2_-induced oxidative stress by reducing ROS and apoptosis and suppressed LPS-induced neuroinflammation in BV-2 microglia via downregulation of iNOS and COX-2. These results suggest that Parishin B is a chemically traceable constituent with dual neuroprotective actions, validating the docking-guided screening strategy and supporting further investigation of *G. elata* as a source of AD-relevant bioactive compounds.

## Simple Summary

Neuroinflammation is an active focus in Alzheimer’s disease (AD) research. Using PDE4B, an enzyme that promotes inflammatory responses in microglia, as a screening target, we analyzed the water extract of *Gastrodia elata* and identified Parishin B as a prioritized constituent. In zebrafish larvae with aluminum-induced behavioral deficits, Parishin B improved swimming activity and blue-zone residence; these measures showed no statistically significant difference from the donepezil-treated group under the present experimental conditions. In cultured SH-SY5Y cells, Parishin B reduced oxidative damage and apoptosis, and in BV-2 microglia, it suppressed LPS-induced inflammatory markers including NO, iNOS, and COX-2. The crude extract also showed protective effects in the same zebrafish model. These results identify Parishin B as a chemically traceable lead for mechanistic follow-up and illustrate a target-anchored screening approach for prioritizing active constituents from herbal extracts.

## 1. Introduction

Alzheimer’s disease (AD), the most common neurodegenerative disorder worldwide, is characterized by progressive memory loss, cognitive impairment, and behavioral disturbances. Its pathogenesis is complex, involving β-amyloid (Aβ) deposition, abnormal tau phosphorylation, neuroinflammation, and oxidative stress [1]. As the global population ages, the incidence of AD continues to rise, making it a pressing public health challenge [2,3]. Current first-line therapies are mainly cholinesterase inhibitors; although drugs such as donepezil hydrochloride can partially improve cognition, they act on a single target, show large inter-individual variability in long-term efficacy, and cannot reverse neurodegeneration [4,5]. Identifying safe, multi-target active constituents from natural products therefore remains an important direction in AD drug discovery.

The medicinal use of *Gastrodia elata* Blume dates back more than 2000 years, with records in ancient Chinese materia medica for treating neurological symptoms [6]. Modern studies have identified diverse constituents in *G. elata*, including gastrodin, parishins, and 4-hydroxybenzyl alcohol, with reported neuroprotective, anti-inflammatory and antioxidant activities [7,8,9]. Gastrodin enhances Aβ clearance via P-glycoprotein regulation [10], *G. elata* water extract (GWE) improves cognition in AD mice by modulating the gut microbiota [11], and *G. elata* polysaccharides clear Aβ deposits and protect neurons [12]. These findings suggest that *G. elata* acts through multiple pathways, but the specific constituents responsible for its anti-AD effects and their direct molecular targets remain undefined.

Phosphodiesterase 4B (PDE4B) is a key regulatory node in AD-related neuroinflammation. Its aberrant activation lowers cerebral cyclic adenosine monophosphate (cAMP), exacerbating microglial activation, pro-inflammatory cytokine release, and synaptic loss, and thereby aggravating neuronal damage [13]. Early inhibition of PDE4B significantly improves cognition and reduces neuroinflammation in amyloid precursor protein (APP) knock-in and 3xTg-AD mouse models [14,15]. In AD, microglial inflammation and neuronal oxidative injury are not isolated events but form a self-reinforcing cycle that drives disease progression [16]. Accordingly, lipopolysaccharide (LPS)-stimulated BV-2 microglia robustly recapitulate key features of microglial activation and neuroinflammation and are widely used for anti-inflammatory evaluation [17], whereas SH-SY5Y neuroblastoma cells are a widely used neuronal model that reproduces AD-relevant insults such as oxidative injury and are convenient for probing neuroprotective effects [18,19].

Although the anti-AD potential of *G. elata* and its constituents has been preliminarily demonstrated [12,20,21], clear gaps remain: the constituents in *G. elata* water extract with high affinity for PDE4B have not been systematically prioritized; the in vivo anti-AD activity and dose–response relationship are insufficiently characterized; and the in vitro protective actions within the interacting oxidative-stress and neuroinflammation microenvironment are unclear. Guided by this rationale, and because direct affinity-based fishing of binders from a complex herbal matrix is often confounded by co-eluting and high-abundance components, we adopted a chemical-profiling-guided, docking-then-validation strategy, as exemplified by recent docking-based screening of phytochemicals against AD-related targets [22], in which PDE4B served not as a presupposed sole target but as a rational molecular anchor linking the traditional anti-forgetfulness use of *G. elata* to a tractable modern mechanism. Specifically, we first characterized the main constituents of *G. elata* water extract by UPLC-ESI-Q-Orbitrap MS; used molecular docking against two PDE4B conformations (PDB IDs: 4MYQ and 1XMY) merely as a computational funnel to prioritize candidates; and experimentally confirmed direct PDE4B inhibition of the top candidate, Parishin B, using a recombinant-enzyme assay. We then verified anti-AD efficacy at two levels—the whole extract and purified Parishin B—in an Al^3+^-induced zebrafish model, which offers high throughput, low cost, and amenability to rapid behavioral screening [23,24] and has also been shown to recapitulate some aspects of AD-like Aβ and tau pathology [25,26]. Consistent with this, a recent study using a plus-maze color preference assay reported that Al^3+^-induced AD zebrafish larvae show a significant reduction in blue-zone swimming distance, accompanied by Aβ_1–42_ deposition and impaired locomotor capacity [27]. Finally, we dissected the underlying mechanisms along two complementary axes: PDE4B/cAMP-associated anti-neuroinflammation (LPS-induced BV-2 model) and general neuroprotection against oxidative injury (H_2_O_2_-induced SH-SY5Y model). This strategy is intended not only to explain, at the whole-herb level, the traditional medicinal use of *G. elata*, but also to chemically track a developable representative constituent from it.

## 2. Materials and Methods

### 2.1. Materials and Instruments

*Gastrodia elata* Blume was purchased from the Anguo Medicinal Material Market (Anguo, China) and authenticated by one of the authors (Nankai University; voucher specimen No. GE202508). Parishin B (HPLC purity ≥ 98%) was obtained from Chengdu Aibo Ke Biotechnology Co., Ltd. (Chengdu, Sichuan, China); donepezil hydrochloride from Shanghai Dibai Biotechnology Co., Ltd. (Shanghai, China); and aluminum chloride (AlCl_3_) from Anhui Zesheng Technology Co., Ltd. (Anqing, China). Chromatographic-grade methanol, acetonitrile, and formic acid were purchased from Fisher Scientific (Pittsburgh, PA, USA) and Tianjin Obt Chemical Co., Ltd. (Tianjin, China); analytical-grade ethanol was from Tianjin Jiangtian Chemical Technology Co., Ltd. (Tianjin, China). Ultrapure water was prepared with a Millipore Direct-Q3 system (Millipore, Burlington, MA, USA). SH-SY5Y human neuroblastoma cells and BV-2 microglia were obtained from the Cell Bank of the Chinese Academy of Sciences (Shanghai, China).

The instruments used were a Vanquish UHPLC system and an Orbitrap Exploris 120 high-resolution mass spectrometer (both Thermo Fisher Scientific, Waltham, MA, USA); a TGL-16 high-speed refrigerated centrifuge (Yongkang Bo’ou Hardware Products Co., Ltd., Yongkang, China); an RE-201D rotary evaporator (Gongyi Yuhua Instrument Co., Ltd., Gongyi, China); a 600Y grinder (Yongkang Bo’ou Hardware Products Co., Ltd., Yongkang, China); an SPX-150B biochemical incubator (Tianjin Hongnuo Instrument Co., Ltd., Tianjin, China); an MK3 microplate reader (Thermo Fisher Scientific, Waltham, MA, USA); an LSR Fortessa flow cytometer (BD Biosciences, San Jose, CA, USA); a Tanon 5200 Multi chemiluminescence imaging system (Shanghai Tianneng Technology Co., Ltd., Shanghai, China); and a DanioVision high-throughput zebrafish behavior analysis system with EthoVision XT 11.5 software (Noldus Information Technology, Wageningen, The Netherlands).

### 2.2. Preparation of Gastrodia elata Water Extract

Dried *G. elata* (50.0 g) was coarsely crushed and then ground with a grinder (15 s per cycle, two cycles). The material was transferred to a 1000 mL round-bottom flask, 500 mL of ultrapure water was added, and reflux extraction was performed at 100 °C for 40 min. The mixture was filtered while hot, and the residue was extracted a second time with 500 mL of ultrapure water at 100 °C for 30 min. The two filtrates were combined and filtered hot to remove residual solids. The filtrate was allowed to stand at room temperature for 2 h, and the supernatant was collected; the remaining precipitate was centrifuged at 5000 rpm for 15 min, and this supernatant was combined with the standing supernatant to give the total extract, which was concentrated under reduced pressure to approximately 100 mL.

The concentrate was placed in a beaker, and anhydrous ethanol was added slowly under magnetic stirring (200 rpm) to a final ethanol volume fraction of 80%. After a further 10 min of stirring, the beaker was sealed and kept at 4 °C for 12 h for ethanol precipitation. After precipitation, the supernatant was collected, concentrated under reduced pressure to a residue, redissolved in ultrapure water with ultrasonication, and lyophilized; the lyophilized powder was then weighed and used for zebrafish behavioral experiments. For UPLC-MS analysis, 1 mL of the same supernatant was gently aspirated, purified through a DDS C18 solid-phase extraction cartridge, and centrifuged at 12,000 rpm and 4 °C for 10 min. The resulting supernatant was passed through a 0.22 μm organic-phase membrane filter, and the filtrate was sealed and stored at 4 °C until analysis.

### 2.3. UPLC-ESI-Q-Orbitrap MS Analysis

Chromatographic conditions. Separation was performed on a Vanquish UHPLC system with an ACQUITY UPLC HSS T3 column (1.8 μm, 2.1 mm × 100 mm; Waters Corporation, Milford, MA, USA) at 35 °C. The injection volume was 2 μL and the flow rate was 0.3 mL/min. The mobile phase consisted of 0.1% aqueous formic acid (A) and acetonitrile (B), with the following gradient: 0–5 min, 2% B; 5–38 min, 2–40% B; 38–45 min, 40–75% B; 45–48 min, 75–98% B; 48–54 min, 98% B; 54–57 min, 98–2% B; and 57–60 min, 2% B.

Mass spectrometry conditions. Detection used an Orbitrap Exploris 120 mass spectrometer with a heated electrospray ionization (HESI) source. Nitrogen served as the nebulizing, sheath, and auxiliary gas; the spray voltage was 3.0 kV in both positive and negative modes; the sheath and auxiliary gas flow rates were 35 and 10 L/h, respectively; the capillary temperature was 320 °C; and the auxiliary heater temperature was 350 °C. Data were acquired in positive and negative full-scan mode over *m*/*z* 80–1200 (full-scan resolution, 120,000; automatic gain control set to automatic; MS^2^ resolution, 15,000), with stepped higher-energy collisional dissociation (HCD) energies of 20/40/60 V.

### 2.4. Molecular Docking

Structures of *G. elata* constituents were retrieved from the PubChem and SciFinder databases and energy-minimized in Chem3D 21.0.0 using the MM2 force field. Target crystal structures were downloaded from the RCSB PDB. PDE4B adopts open (1XMY) and closed (4MYQ) conformations during cAMP hydrolysis; because docking against a single conformation risks high false-negative rates [28], both structures were used to reduce missed hits and to assess conformational adaptability. In addition, closed-state binding engages the PDE4B-specific residue Leu674 [29], which favors preferential PDE4B inhibition and may mitigate the emesis associated with PDE4D suppression [30]. Proteins and ligands were prepared in AutoDockTools 1.5.7: water molecules and co-crystallized ligands were removed, polar hydrogens and Kollman charges were added to the protein, protonation states were assigned using default settings, Gasteiger charges were assigned to the energy-minimized ligands, and both were saved in PDBQT format.

Docking was performed with AutoDock Vina 1.1.2, and the binding-pocket parameters were defined from the co-crystallized ligand of each structure using the Getbox-PyMOL-Plugin (developed by Professor Mengwu Xiao’s group, Hunan University). For 4MYQ, the pocket was centered at (−16.9, 34.6, −6.6) with dimensions 17.3 × 21.1 × 15.6 Å; for 1XMY, it was centered at (43.4, 1.7, 64.5) with dimensions 18.8 × 13.6 × 17.3 Å. The exhaustiveness was set to 8 (default), and the energy range was 3 kcal/mol (default). Nine conformations were generated per ligand–protein complex, ranked by binding energy, and the top conformation of each ligand was visualized in PyMOL 2.6.0a0. Polar contacts were identified using the “Find polar contacts” function in PyMOL (Action → Find → Polar contacts → to any atoms) with default geometric criteria (h_bond_cutoff_center = 3.6 Å, h_bond_cutoff_edge = 3.2 Å). Docking scores were used only for relative prioritization among candidates, not as absolute measures of binding affinity. To assess reliability, each co-crystallized ligand was re-docked into its native pocket under identical parameters, and the root-mean-square deviation (RMSD) between the experimental and predicted poses was calculated; an RMSD below 2.0 Å was considered reliable.

### 2.5. PDE4B Enzymatic Inhibition Assay

The effect of Parishin B on PDE4B activity was assessed with a fluorescence-polarization–based PDE4B (rat) assay kit (BPS Bioscience, San Diego, CA, USA, Cat. No. 79571). Recombinant rat PDE4B was incubated with Parishin B (1, 10, or 100 μM) or roflumilast (0.1 nM, positive control) in the presence of 200 nM FAM-cyclic-3′,5′-AMP substrate for 1 h at room temperature. After addition of the PDE binding agent, fluorescence polarization (mP) values were recorded. Each run included a reference control (substrate only, no enzyme) and a positive control (enzyme and substrate, no inhibitor). Residual activity was calculated as:

$$\%\;\mathrm{of Activity}=\frac{\left(\mathrm{mP value from Test Inhibitor}-\mathrm{mP value from Reference Control}\right)}{\left(\mathrm{mP value from Positive Control}-\mathrm{mP value from Reference Control}\right)}\times 100$$

Each concentration was tested in three independent experiments (n = 3), and data are expressed as the mean ± standard deviation (SD).

### 2.6. In Vivo Efficacy in a Zebrafish AD Model

#### 2.6.1. Animals

Adult AB wild-type zebrafish were purchased from Shanghai Feixi Biotechnology Co., Ltd. and maintained in a recirculating system at 28.5 ± 0.5 °C with continuous aeration and ultraviolet disinfection. Fish were fed brine shrimp nauplii twice daily under a 14 h light/10 h dark photoperiod. All procedures complied with the regulations of the Experimental Animal Ethics Committee of Nankai University (approval No. 2021-SYDWLL-000225).

#### 2.6.2. Embryo Collection

The night before spawning, healthy sexually mature males and females were placed in four mating tanks at a 1:2 (male:female) ratio (eight females and four males), separated by a transparent baffle, and kept overnight at 28.5 ± 0.5 °C in the dark. The next morning, the baffle was removed after 1 h of light, and embryos were collected 2 h later. Embryos from the four tanks were pooled, and healthy embryos at 6 h post-fertilization (hpf) were divided into six groups (30 embryos per group) and cultured in Holt buffer (HB) at 28.5 °C under a 14 h light/10 h dark photoperiod.

#### 2.6.3. Effect of *G. elata* Water Extract on AlCl_3_-Induced Behavioral Changes

Healthy larvae at 72 hpf were randomly assigned to six groups (n = 30 per group): blank control, AlCl_3_ model, AlCl_3_ + donepezil hydrochloride (DPZ), and AlCl_3_ + *G. elata* water extract (GWE) at 10, 30, and 100 μg/mL. The blank control was maintained in HB throughout. The AlCl_3_ model group was exposed to 100 μM AlCl_3_ from 72 to 120 hpf (48 h), then washed with HB and cultured until testing. The DPZ and GWE groups were first exposed to 100 μM AlCl_3_ until 120 hpf, washed, and then treated for a further 48 h with 10 μM DPZ (≈4.2 μg/mL) or the corresponding GWE concentrations, respectively. After treatment (168 hpf), and based on the reported innate preference of zebrafish for blue in a water-maze paradigm [31,32], six surviving larvae per group (n = 6) were each observed for 10 min. Each larva was tested individually in one well of a six-well plate using the DanioVision system at a frame rate of 25 frames per second. Behavioral data were recorded automatically by the system, and the center-point of each larva was tracked. The test arena was a cross-shaped maze with a diameter of 24 mm, divided into five interconnected zones of approximately 64 mm^2^ each. The central zone was colorless, while the four outer zones were colored blue (top), red (left), green (right), and yellow (bottom). The assay was performed under uniform overhead lighting, and each larva was allowed to acclimate for 60 s before the 10-min recording. Color positions were fixed and not counterbalanced. Total swimming distance, mean swimming speed, and cumulative residence time in the blue target zone were recorded, and trajectory maps and heatmaps were generated. The sample size was based on power calculations for large effect sizes [33] and is consistent with previous zebrafish AD studies [34].

#### 2.6.4. Effect of Parishin B on AlCl_3_-Induced Behavioral Changes

The safe concentration of Parishin B was first evaluated in a biocompatibility test. Healthy AB embryos at 6 hpf were randomly divided into groups in six-well plates (30 embryos per well; 4 mL exposure volume): a blank control in HB and treatment groups in HB containing 15, 30, 60, 90, or 120 μM Parishin B. Exposures were conducted at 28.5 ± 0.5 °C under a 14 h light/10 h dark photoperiod using a semi-static method, with the exposure solution renewed every 24 h until 78 hpf; mortality and abnormal phenotypes were recorded every 24 h. The behavioral protocol followed Section 2.6.3, except that the treatment groups were AlCl_3_ + Parishin B (10, 30, and 100 μM), with all other conditions held constant.

### 2.7. In Vitro Mechanistic Assays

#### 2.7.1. Cell Culture

SH-SY5Y and BV-2 cells were cultured in complete medium (Dulbecco’s Modified Eagle Medium [DMEM] containing 1% penicillin–streptomycin and fetal bovine serum at a 9:1 volume ratio) at 37 °C under 5% CO_2_.

#### 2.7.2. Cell Viability Assay

Cell viability was assessed by the 3-(4,5-dimethylthiazol-2-yl)-2,5-diphenyltetrazolium bromide (MTT) assay [35] to determine the safe concentration of Parishin B. SH-SY5Y cells (4 × 10^4^ per well) and BV-2 cells (2 × 10^4^ per well) were seeded in 96-well plates and cultured for 24 h. Parishin B was then added at 1, 3, 10, 30, and 100 μM, and cells were cultured for a further 24 h. MTT solution (20 μL) was added and incubated for 4 h; the medium was removed, 150 μL of dimethyl sulfoxide was added per well, and absorbance was measured at 492 nm.

#### 2.7.3. Protection Against H_2_O_2_-Induced Oxidative Injury

To establish the model, SH-SY5Y cells (4 × 10^4^ per well) were seeded in 96-well plates, cultured for 24 h, treated with graded H_2_O_2_ concentrations for 24 h, and assessed by MTT to determine the modeling concentration.

Viability. Parishin B (16, 40, or 100 μM) or epigallocatechin gallate (EGCG, 30 μM; positive control) was applied 30 min before adding 900 μM H_2_O_2_ [36], and viability was measured after 24 h of co-incubation.

Apoptosis. SH-SY5Y cells (3 × 10^5^ per well) were seeded in 12-well plates and cultured for 24 h. Parishin B (16, 40, or 100 μM) was added 30 min before H_2_O_2_, and cells were co-treated for 24 h. Cells were collected, stained with Annexin V-FITC and propidium iodide (PI) for 15 min per the apoptosis kit instructions, passed through a 200-mesh sieve, and analyzed by flow cytometry [37] using FlowJo 7.6.1.

ROS. SH-SY5Y cells (3 × 10^5^ per well) were treated as above. Following the ROS assay kit instructions [38], cells were collected, and the DCFH-DA probe (diluted to 10 μM in pre-cooled serum-free DMEM) was added and incubated at 37 °C for 20 min. After washing, cells were resuspended in pre-cooled serum-free DMEM, sieved, and kept on ice in foil-wrapped tubes. The mean fluorescence intensity (MFI) in the FITC channel, reflecting intracellular ROS, was measured by flow cytometry and analyzed in FlowJo.

#### 2.7.4. Protection Against LPS-Induced Neuroinflammation

NO release. BV-2 cells in the exponential phase (2 × 10^4^ per well) were seeded in 96-well plates and cultured for 24 h, then stimulated with 0.2 μg/mL LPS. Parishin B (16, 40, or 100 μM) was added, with S-methylisothiourea (SMT) as a positive control. After 24 h of co-treatment, 50 μL of supernatant was mixed with 50 μL of Griess reagent (Griess A:Griess B = 1:1), the optical density was read at 550 nm, and NO was calculated from a standard curve [39].

Western blot. BV-2 cells in the exponential phase (6 × 10^6^ per well) were seeded in six-well plates. After treatment, cells were washed twice with pre-cooled phosphate-buffered saline (PBS) and lysed in RIPA buffer containing 1 mM phenylmethylsulfonyl fluoride (PMSF). Lysates were rotated at 4 °C for 30 min and centrifuged at 12,000 rpm and 4 °C for 10 min; the supernatant served as the protein sample [40]. Protein concentration was determined by the bicinchoninic acid (BCA) method. Samples were mixed with loading buffer, denatured at 100 °C, and stored at −80 °C. Proteins were separated by sodium dodecyl sulfate–polyacrylamide gel electrophoresis (SDS-PAGE), transferred to polyvinylidene fluoride (PVDF) membranes at constant current, blocked with 5% skim milk for 1.5 h, and incubated overnight at 4 °C with primary antibodies (β-actin, iNOS, COX-2). Membranes were washed with Tris-buffered saline containing Tween 20 (TBST; six times, 5 min each), incubated with secondary antibody for 1 h, and washed again with TBST (30 min total, 5 min each). Bands were visualized by chemiluminescence and quantified in ImageJ 1.51k.

### 2.8. Statistical Analysis

For in vivo behavioral experiments, six larvae per group were tested (n = 6), and data are expressed as the mean ± SD. Analysis used one-way analysis of variance (ANOVA) followed by the LSD or Dunnett’s T3 post hoc test in IBM SPSS Statistics 26; bar graphs were prepared in OriginPro 2021 (version 9.8.0.200). For in vitro experiments, all assays were performed in three independent replicates (n = 3), and data are expressed as the mean ± SD. Analysis used one-way ANOVA followed by Tukey’s multiple-comparison test in GraphPad Prism 6.0. A value of *p* < 0.05 was considered statistically significant.

## 3. Results

### 3.1. Chemical Profiling of G. elata Water Extract

Mass spectrometric data were processed in Thermo Scientific Xcalibur 4.1.50 (Thermo Fisher Scientific, Waltham, MA, USA). The molecular formula of each constituent was predicted from the accurate mass of the quasi-molecular ion in positive and negative modes and then matched against an in-house database together with MS^2^ fragments, yielding 17 major constituents. Several peaks were tentatively assigned to isomeric pairs (Parishin B/C, E/G, I/V, and H/M) under the current LC-MS conditions (see Table 1 footnote).

**Figure 1:**
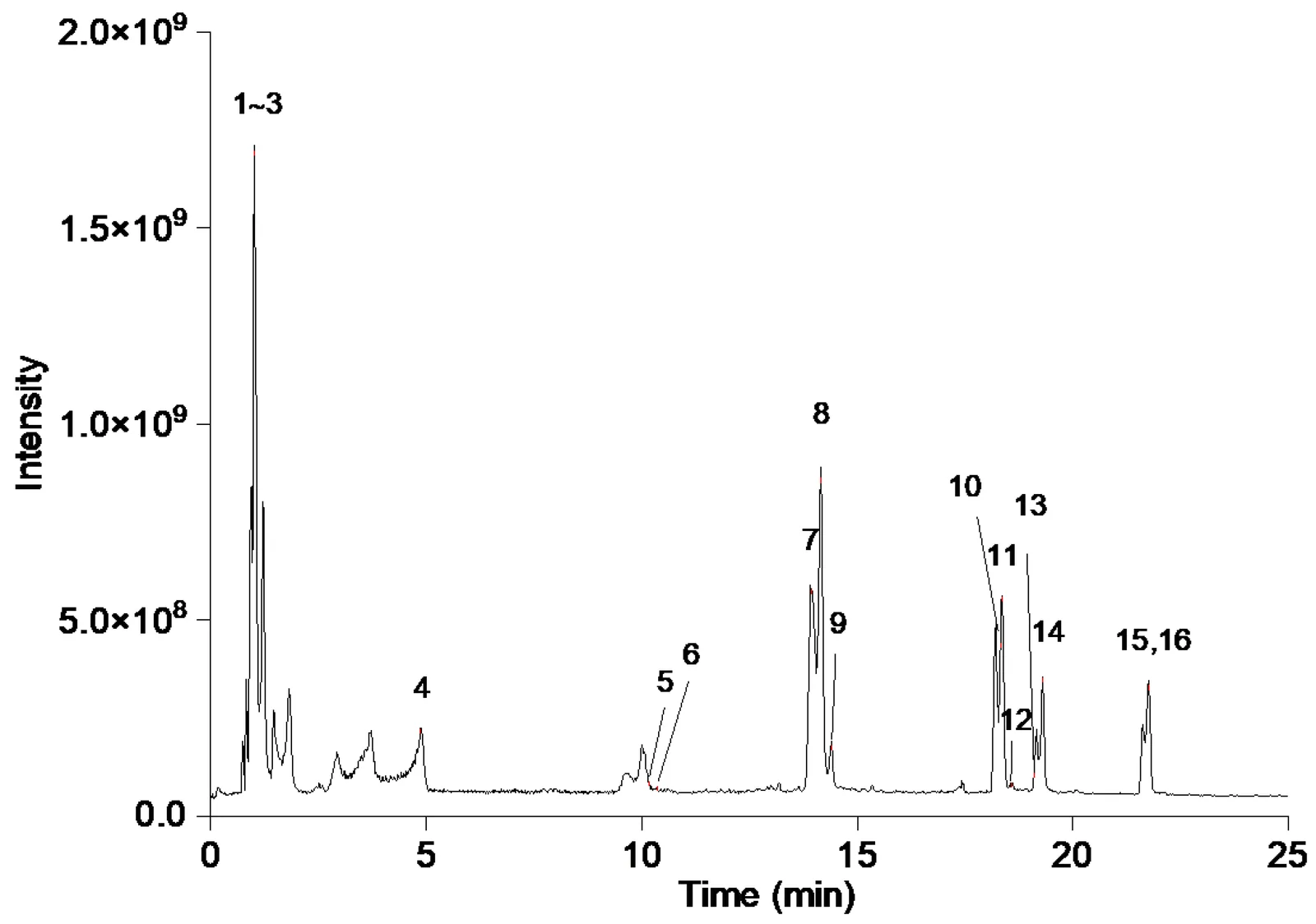
Total ion chromatogram of *G. elata* water extract in negative mode (0–25 min). The peaks correspond to the 17 main chemical components identified in Table 1. Through high-resolution mass spectrometry analysis, the 13th chromatographic peak could not distinguish two isomers due to co-elution. Combined with accurate molecular weight and fragment information, it was assigned to two possible components, and a total of 17 compounds were identified.

With the 60 min method, the target constituents eluted mainly within 0–25 min, and no notable chromatographic peaks or mass signals were detected thereafter. Therefore, only the 0–25 min segment of the negative-mode total ion chromatogram was shown (Figure 1), with detailed information for each constituent in Table 1. Chromatographic peak 13 could not be resolved into its two co-eluting isomers; based on accurate mass and fragmentation, it was assigned to two possible constituents, giving 17 compounds in total.

### 3.2. Docking-Guided Prioritization of Parishin B as a Candidate Targeting PDE4B

To focus on constituents with plausible anti-AD relevance, 14 characteristic compounds were selected from the 17 identified (excluding basic compounds of unclear anti-AD relevance, such as lactobionic acid, sucrose, and isomaltose) for docking. To improve reliability, docking was performed against two PDE4B structures (PDB IDs: 4MYQ and 1XMY). Several constituents bound both structures strongly (binding energies below −7.0 kcal/mol) and interacted with key active-site residues (Figure S1, Table 2); most also interacted with metal ions, including Zn^2+^ and Mg^2+^, indicating a multi-site binding mode.

**Figure 2:**
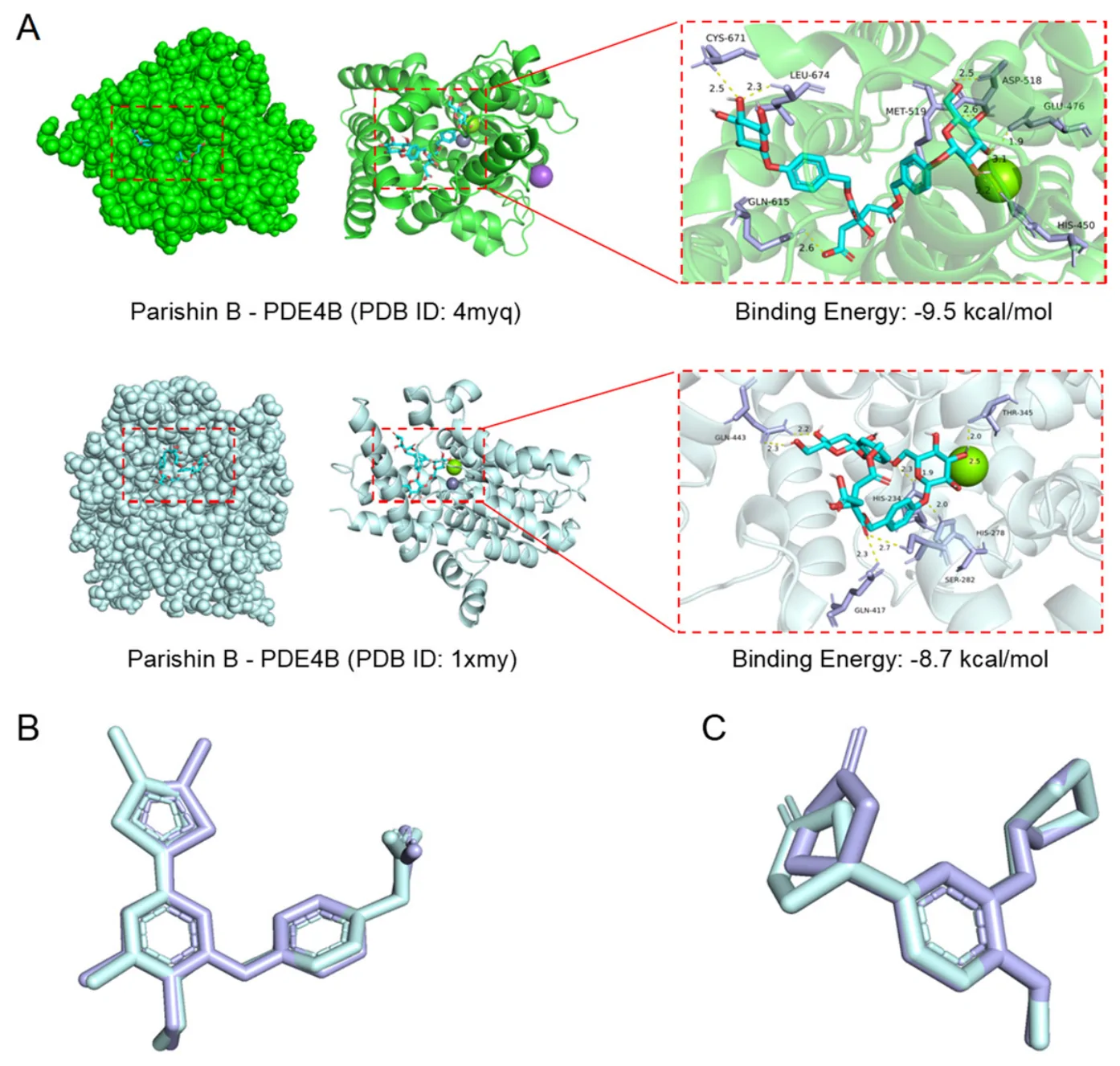
Molecular docking analysis. (**A**) Binding conformations of Parishin B with PDE4B (4MYQ and 1XMY). (**B**) Superposition of re-docked and co-crystallized ligand 19T in PDE4B (4MYQ). (**C**) Superposition of re-docked and co-crystallized ligand ROL in PDE4B (1XMY). Co-crystal ligands are shown as light-blue stick models, and re-docked conformations are shown as pale-cyan stick models. The RMSD values are 0.295 Å and 0.134 Å, respectively (RMSD < 2.0 Å indicates a reliable docking method).

To integrate the dual-structure data, a normalized comprehensive docking score was used to rank overall binding potential (Table S1). Parishin B achieved the highest score (0.9333), with docking energies of −9.5 and −8.7 kcal/mol toward PDE4B (4MYQ) and PDE4B (1XMY), respectively (Figure 2A). It interacted with Mg^2+^ and formed hydrogen bonds with key residues, including Gln615, Met519, and His450 in 4MYQ, and Gln443, Thr345, and His234 in 1XMY, all located within the core functional regions. These results indicated that Parishin B showed predicted favorable binding modes within the PDE4B active site and might modulate its activity, consistent with the established use of docking to prioritize natural compounds as candidate inhibitors [45,46]. Parishin B was therefore prioritized for functional validation.

Re-docking confirmed the reliability of the protocol: the RMSD was 0.295 Å for the 4MYQ ligand 19T and 0.134 Å for the 1XMY ligand ROL (Figure 2B,C), both below the 2.0 Å threshold.

### 3.3. Parishin B Inhibits PDE4B Enzymatic Activity

To determine whether Parishin B directly affects PDE4B catalysis, we performed an orthogonal, fluorescence-polarization–based enzymatic assay. The reference control (no enzyme) and positive control (enzyme, no inhibitor) yielded mean mP values of 105.3 and 212.7, respectively.

**Figure 3:**
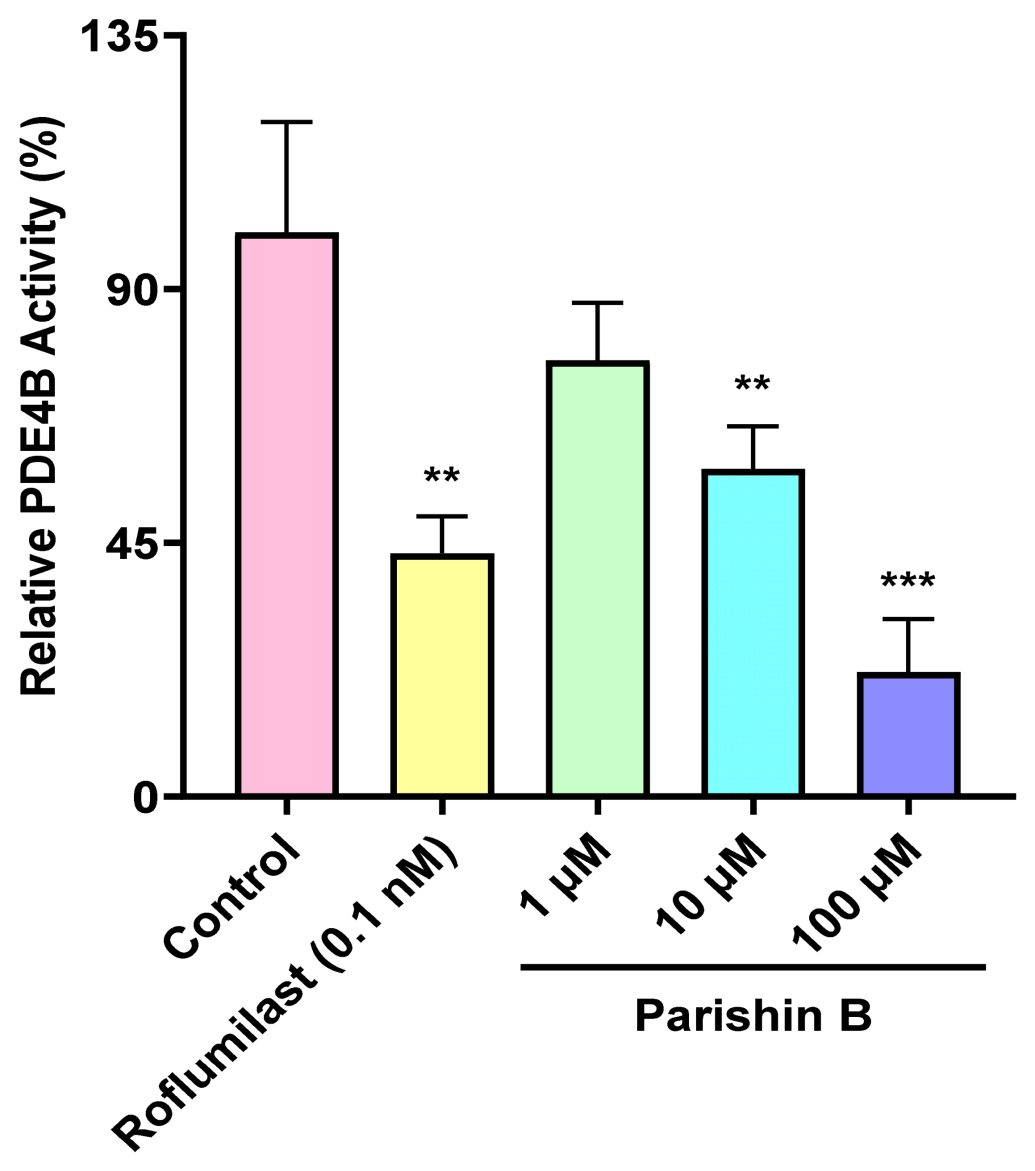
Parishin B inhibits PDE4B enzymatic activity in vitro. PDE4B activity was measured using a fluorescence polarization-based assay (BPS Bioscience #79571) and expressed as percentage of the Positive Control (enzyme without inhibitor, set as 100%). Parishin B was tested at 1, 10, and 100 μM; roflumilast (0.1 nM) was used as a reference inhibitor. Data are expressed as mean ± SD, n = 3. ** *p* < 0.01, *** *p* < 0.001 vs. positive control group.

Parishin B inhibited PDE4B in a concentration-dependent manner (Figure 3), reducing activity to 77.3 ± 10.3%, 58.1 ± 7.6%, and 22.1 ± 9.5% of the positive control at 1, 10, and 100 μM, respectively. Under identical conditions, the reference inhibitor roflumilast (0.1 nM) reduced activity to 43.2 ± 6.5%. These results confirmed that Parishin B directly inhibited PDE4B, consistent with the docking-based prioritization. Raw mP values and calculated % activity for each replicate are provided in Table S2.

### 3.4. In Vivo Efficacy in the Zebrafish AD Model

#### 3.4.1. Anti-AD Activity of *G. elata* Water Extract

**Figure 4:**
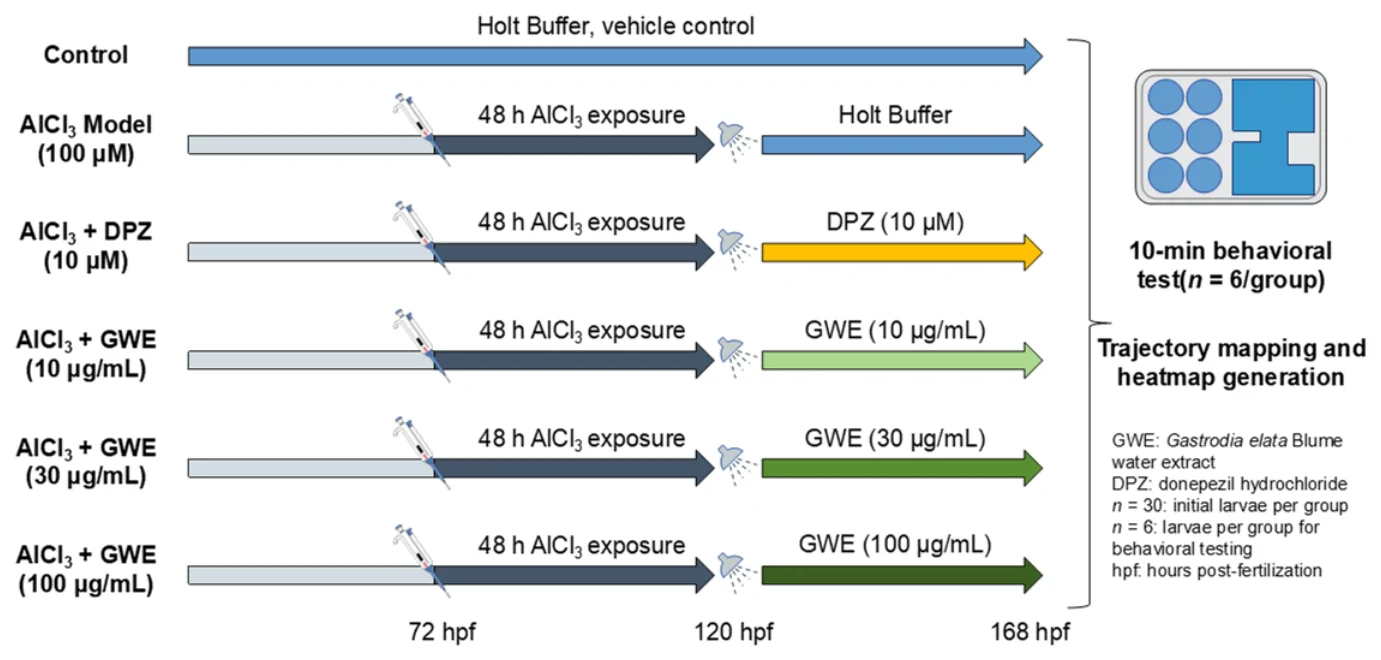
Timeline of experimental design and drug treatment in zebrafish larvae. GWE, *G. elata* water extract; DPZ, donepezil hydrochloride; hpf, hours post-fertilization; n = 30, initial larvae per group; n = 6, larvae per group for behavioral testing.

**Figure 5:**
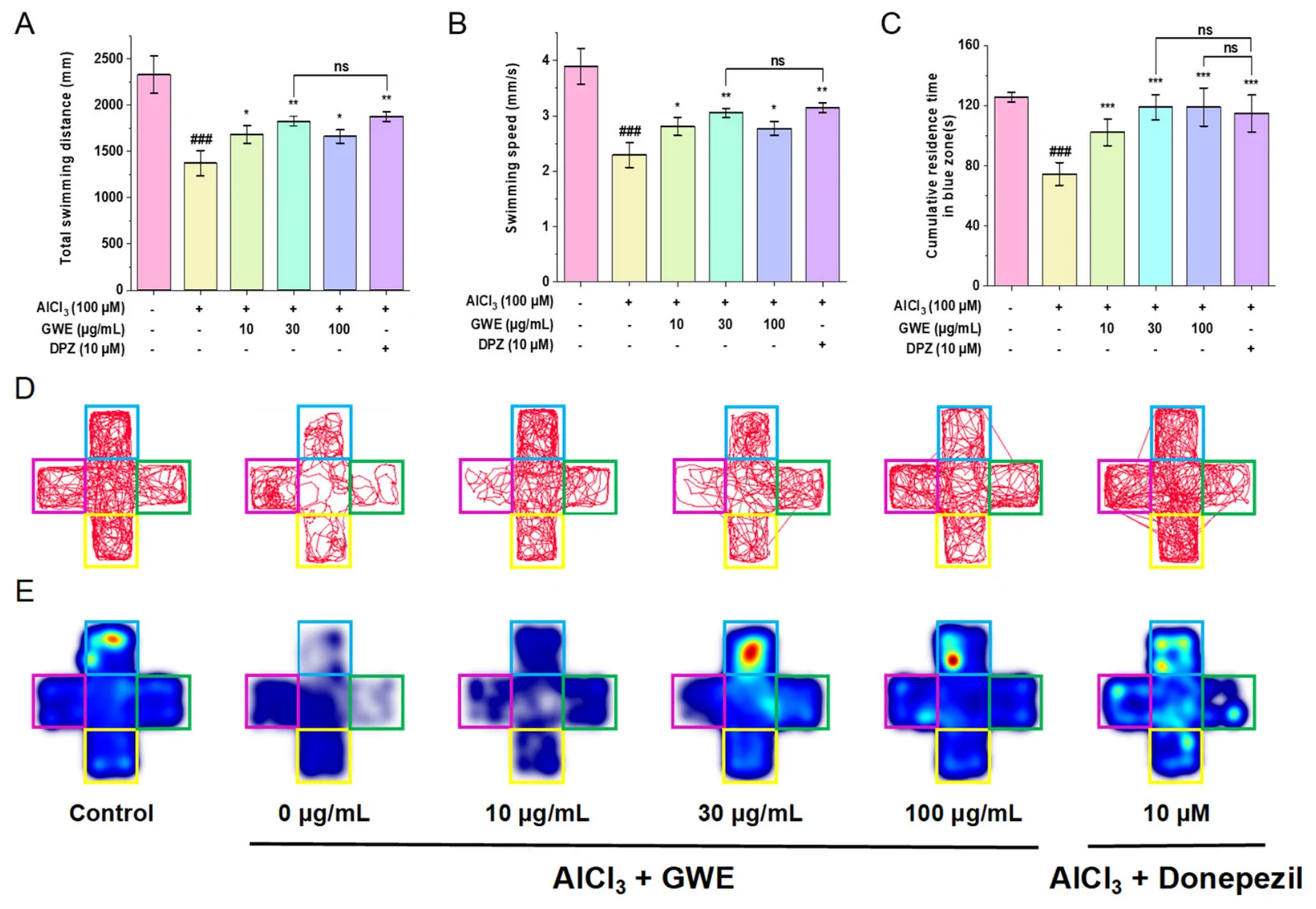
Effect of *G. elata* water extract on AlCl_3_-induced zebrafish behavioral deficits. (**A**) Total swimming distance of zebrafish larvae. (**B**) Changes in swimming speed. (**C**) Cumulative residence time in the blue zone. (**D**) Trajectory diagram. (**E**) Heat map. The colored boxes indicate the four outer zones of the cross-shaped maze: blue (top), red (left), green (right), and yellow (bottom). Representative trajectory diagram (**D**) and heat map (**E**) are from one individual per group and illustrate movement dynamics; quantitative group-level comparisons are shown in panels (**A**–**C**). Data are expressed as mean ± standard deviation (6 larvae per group, independent test). Statistical symbols: ### *p* < 0.001 vs. control group; * *p* < 0.05, ** *p* < 0.01, *** *p* < 0.001 vs. AlCl_3_ model group; ns, no significant difference from the positive drug group.

Thirty normally developed larvae were initially included per group. Survival rates at 168 hpf exceeded 95% in all groups. Six larvae were randomly selected from each group for behavioral testing (n = 6). The experimental timeline was shown in Figure 4. Relative to the blank control, the AlCl_3_ model group showed significantly reduced total swimming distance, mean swimming speed, and residence time in the blue zone (*p* < 0.001; Figure 5), confirming successful model establishment. Compared with the model group, all GWE groups (10, 30, and 100 μg/mL) showed significant recovery of these indices (*p* < 0.05; Figure 5), indicating that GWE effectively ameliorated the AlCl_3_-induced deficits within this range. The 30 μg/mL group showed the greatest improvement, and there was no statistically significant difference in behavior compared with the 10 μM DPZ positive control (*p* > 0.05), whereas increasing the dose to 100 μg/mL produced no further benefit. Complete statistics for all parameters were provided in Table S3, and raw tracking data were provided in Table S4. Together, these results indicated that *G. elata* water extract ameliorated AlCl_3_-induced AD-like behavioral deficits in zebrafish larvae.

#### 3.4.2. Biosafety of Parishin B

**Figure 6:**
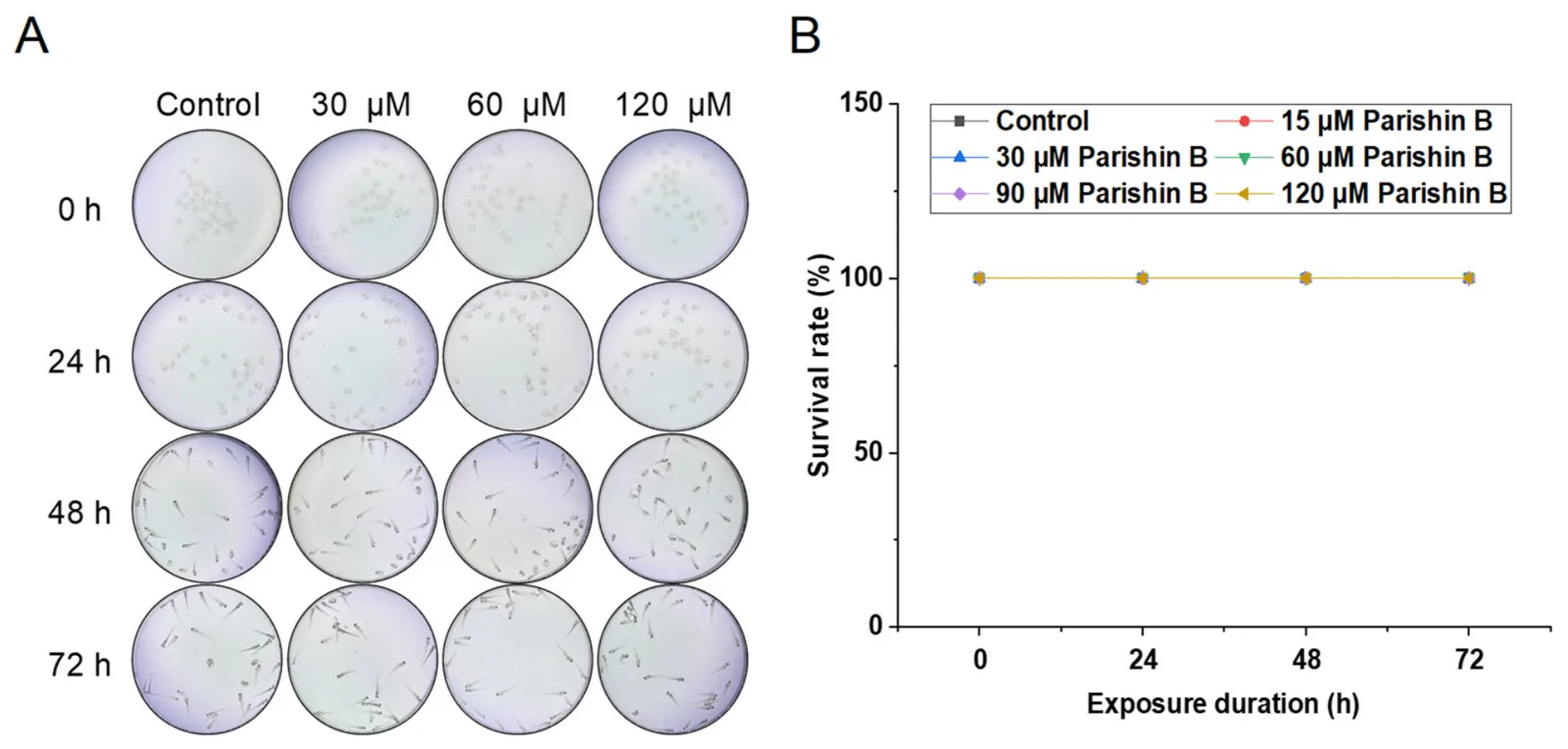
Developmental toxicity of Parishin B in zebrafish embryos. 6 hpf zebrafish embryos were exposed to Parishin B (15, 30, 60, 90, and 120 μM) for 72 h (corresponding to 6–78 hpf). Each group contained 30 embryos, and the assay was performed in a single experiment. (**A**) Representative morphological images. (**B**) Survival rate across all tested concentrations. The survival rates of all groups were 100% during this time period, so the lines overlap completely. The legend is retained to indicate the six groups.

In the biocompatibility test, exposure of 6 hpf embryos to Parishin B at concentrations up to 120 μM for 72 h produced no obvious toxicity (Figure 6), indicating good safety within the range used and providing a basis for the concentrations selected in subsequent behavioral experiments.

#### 3.4.3. Anti-AD Activity of Parishin B

**Figure 7:**
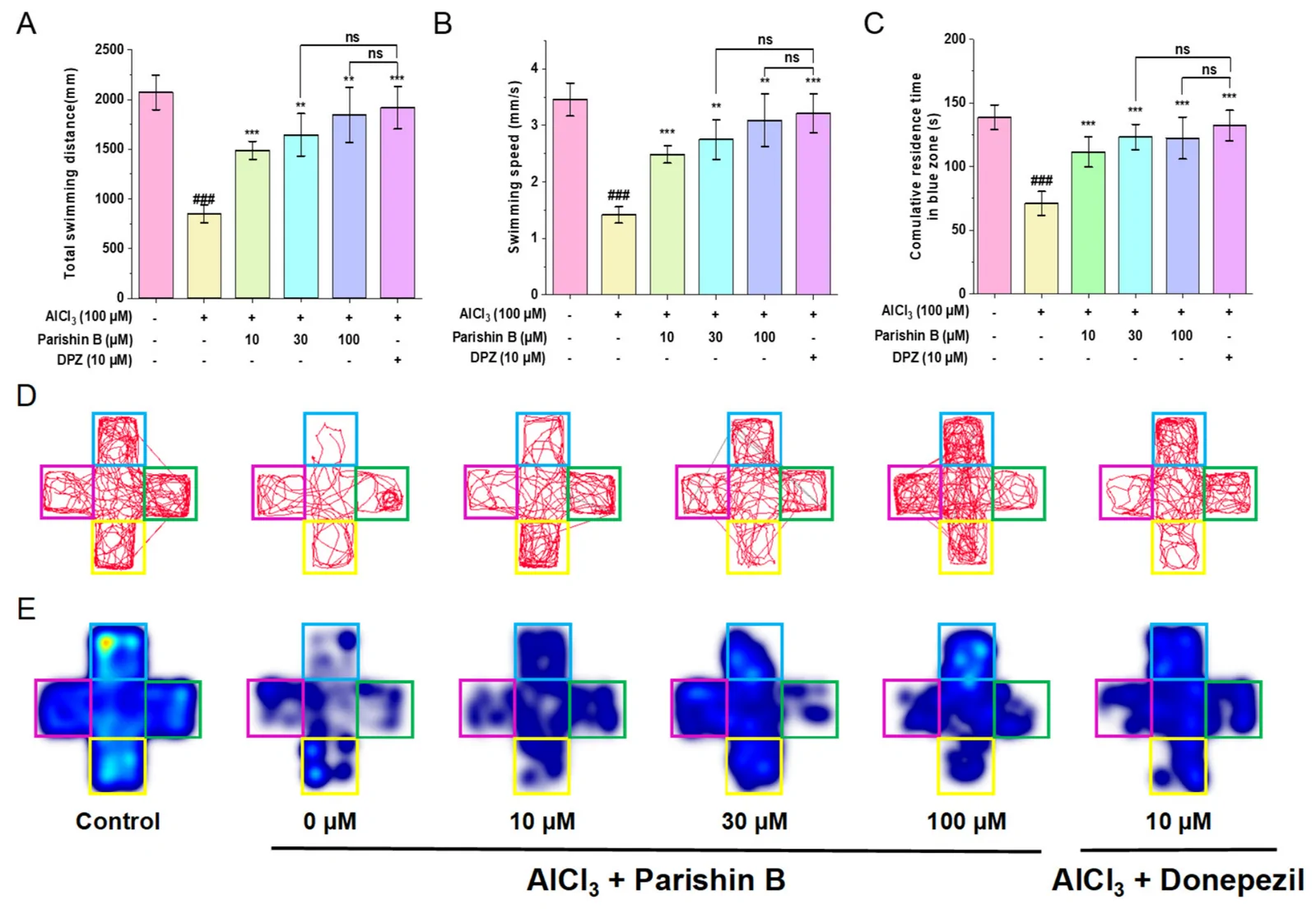
Effect of Parishin B on AlCl_3_-induced zebrafish behavioral deficits. (**A**) Total swimming distance of zebrafish larvae. (**B**) Changes in swimming speed. (**C**) Cumulative residence time in the blue zone. (**D**) Trajectory diagram. (**E**) Heat map. The colored boxes indicate the four outer zones of the cross-shaped maze: blue (top), red (left), green (right), and yellow (bottom). Representative trajectory diagram (**D**) and heat map (**E**) are from one individual per group and illustrate movement dynamics; quantitative group-level comparisons are shown in panels (**A**–**C**). Data are expressed as mean ± standard deviation (6 larvae per group, independent test). Statistical symbols: ### *p* < 0.001 vs. control group; ** *p* < 0.01, *** *p* < 0.001 vs. AlCl_3_ model group; ns, no significant difference from the positive drug group.

Thirty normally developed larvae were initially included per group; after treatment, six surviving larvae per group were randomly selected for testing (n = 6). Relative to the blank control, the AlCl_3_ model group again showed significantly reduced total swimming distance, mean swimming speed, and blue-zone residence time (*p* < 0.001; Figure 7). Compared with the model group, the 10, 30, and 100 μM Parishin B groups showed significant, concentration-dependent recovery (*p* < 0.01; Figure 7). The 100 μM group showed the greatest improvement, and there was no statistically significant difference in behavior compared with the 10 μM DPZ positive control (*p* > 0.05). Complete statistics were provided in Table S5, and raw tracking data were provided in Table S6.

Together, these results showed that both *G. elata* water extract and Parishin B ameliorated AlCl_3_-induced AD-like behavioral deficits in zebrafish larvae, supporting the utility of the docking-guided strategy. Because oxidative stress and neuroinflammation are central to AD and PDE4B is a key regulator of neuroinflammation, we next established an H_2_O_2_-induced SH-SY5Y model and an LPS-induced BV-2 model to define the neuroprotective actions of Parishin B; these two processes interact closely and together drive neuronal loss and cognitive decline in AD [47].

### 3.5. In Vitro Mechanistic Studies

#### 3.5.1. Cytotoxicity of Parishin B in SH-SY5Y and BV-2 Cells

**Figure 8:**
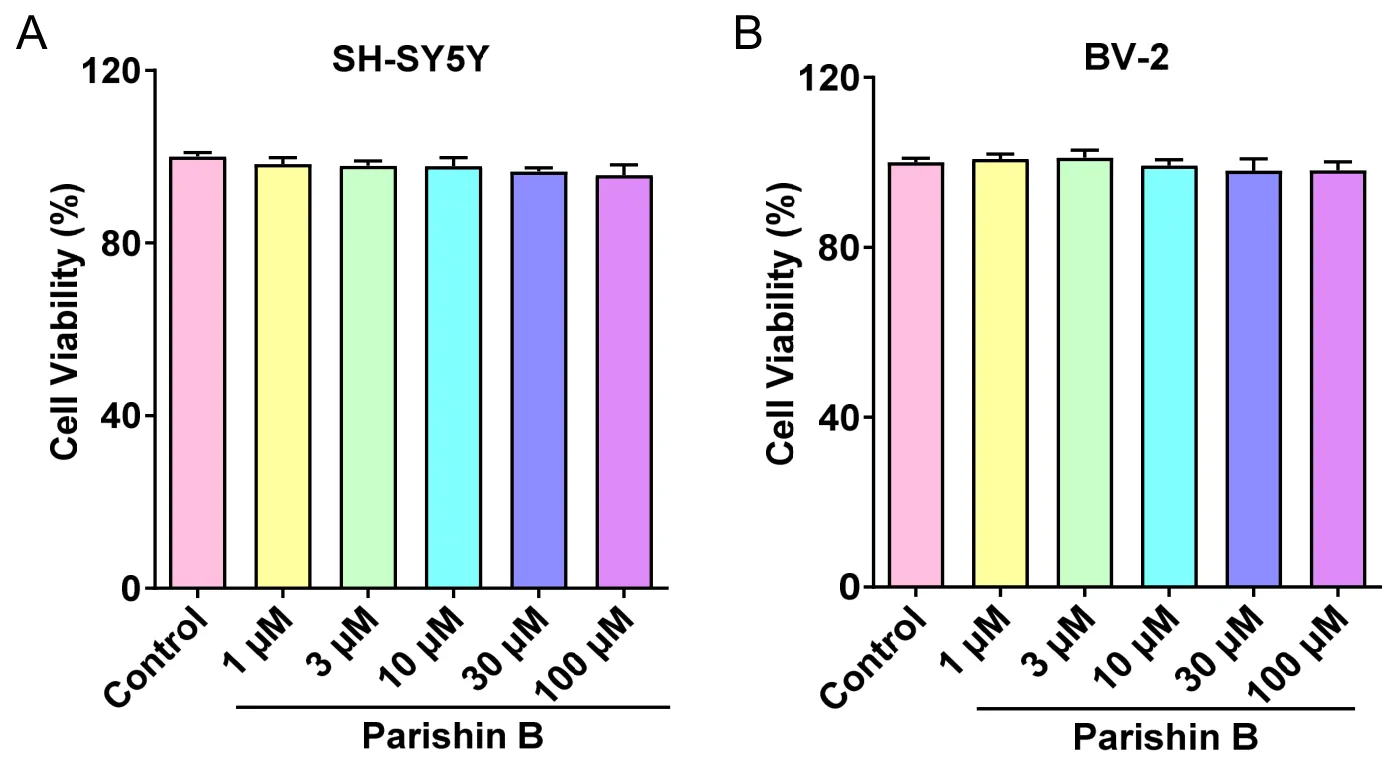
Cytotoxicity of Parishin B in SH-SY5Y and BV-2 cells. (**A**) Effect of Parishin B at different concentrations (1, 3, 10, 30, 100 μM) on the viability of SH-SY5Y cells. (**B**) Effect of Parishin B at different concentrations (1, 3, 10, 30, 100 μM) on the viability of BV-2 cells. Data are expressed as mean ± SD, n = 3. No significant cytotoxicity was observed within the tested concentration range.

By MTT assay, Parishin B (1–100 μM) had no significant effect on the viability of SH-SY5Y (Figure 8A) or BV-2 (Figure 8B) cells, with viability maintained above 90% and no evident cytotoxicity or growth inhibition. Thus, 1–100 μM represented a safe range for subsequent assays.

#### 3.5.2. Neuroprotective Effect of Parishin B Against Oxidative Injury

**Figure 9:**
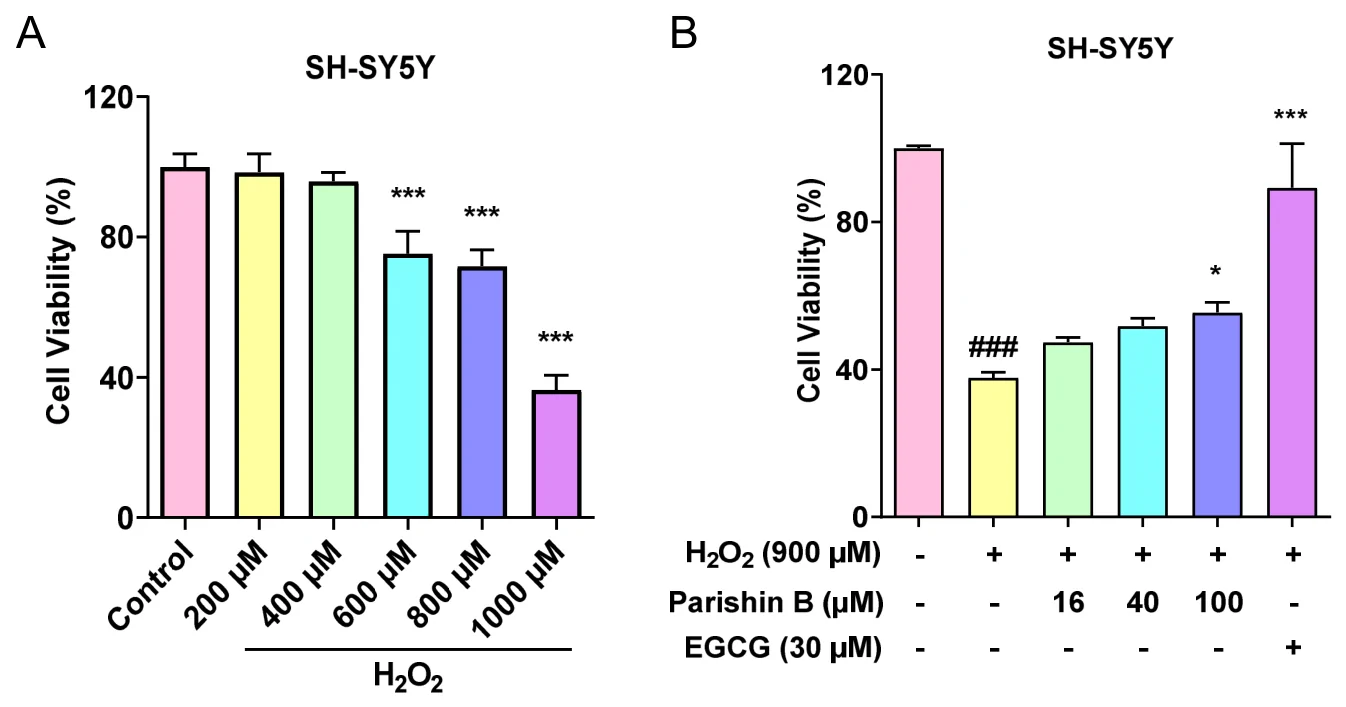
Establishment of oxidative-injury model and protection by Parishin B in SH-SY5Y cells. (**A**) Cytotoxicity of different concentrations of H_2_O_2_ (200, 400, 600, 800, 1000 μM) on SH-SY5Y cells. Based on these results, 900 μM H_2_O_2_ was selected for subsequent experiments. (**B**) Protective effect of Parishin B (16, 40, 100 μM) on 900 μM H_2_O_2_-induced viability loss in SH-SY5Y cells. Epigallocatechin gallate (EGCG, 30 μM) was used as a positive control. Data are expressed as mean ± SD, n = 3. ### *p* < 0.001 vs. control group; * *p* < 0.05, *** *p* < 0.001 vs. model group.

Modeling concentration. After 24 h of treatment with 200–1000 μM H_2_O_2_, SH-SY5Y viability decreased concentration-dependently (Figure 9A); the 600, 800, and 1000 μM groups differed significantly from the control (*p* < 0.001). The IC_50_ fell between 800 and 1000 μM, so 900 μM H_2_O_2_ was selected to achieve approximately 50% injury.

Viability. Compared with the model group, 100 μM Parishin B significantly increased the viability of H_2_O_2_-injured cells (*p* < 0.05; Figure 9B), whereas the 16 and 40 μM groups showed no significant improvement. Thus, at the highest tested concentration, Parishin B protected SH-SY5Y cells against oxidative injury.

**Figure 10:**
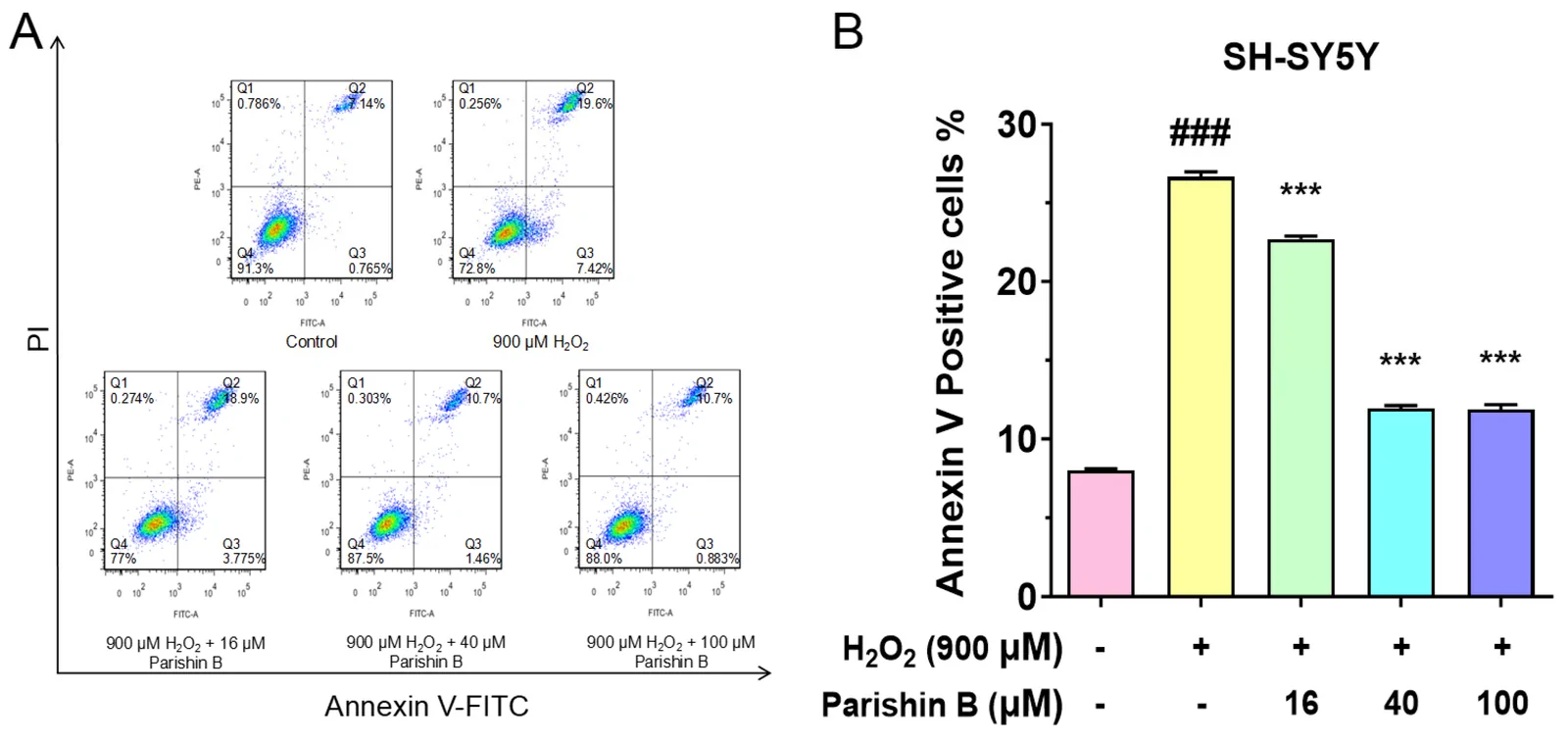
Effect of Parishin B on H_2_O_2_-induced apoptosis in SH-SY5Y cells. (**A**) Scatter plots of Annexin V-FITC/PI double staining flow cytometry. The color gradient from blue to red indicates cell density (blue, low; red, high). (**B**) Quantitative analysis of total apoptosis rate of cells in each group. Control group: normally cultured cells; Model group: treated with 900 μM H_2_O_2_ for 24 h; Parishin B pre-treatment group: pre-treated with Parishin B (16, 40, 100 μM) for 30 min, then added to 900 μM H_2_O_2_ for co-incubation for 24 h. Data are expressed as mean ± SD, n = 3, ### *p* < 0.001 vs. control group; *** *p* < 0.001 vs. model group.

Apoptosis. By Annexin V-FITC/PI staining, the total apoptosis rate was 8.01 ± 0.08% in the control group and increased significantly to 26.67 ± 0.26% after 900 μM H_2_O_2_ (Figure 10A). Pretreatment with 16, 40, and 100 μM Parishin B concentration-dependently reversed H_2_O_2_-induced apoptosis (Figure 10B), indicating that inhibition of apoptosis contributed to its protection against oxidative injury.

**Figure 11:**
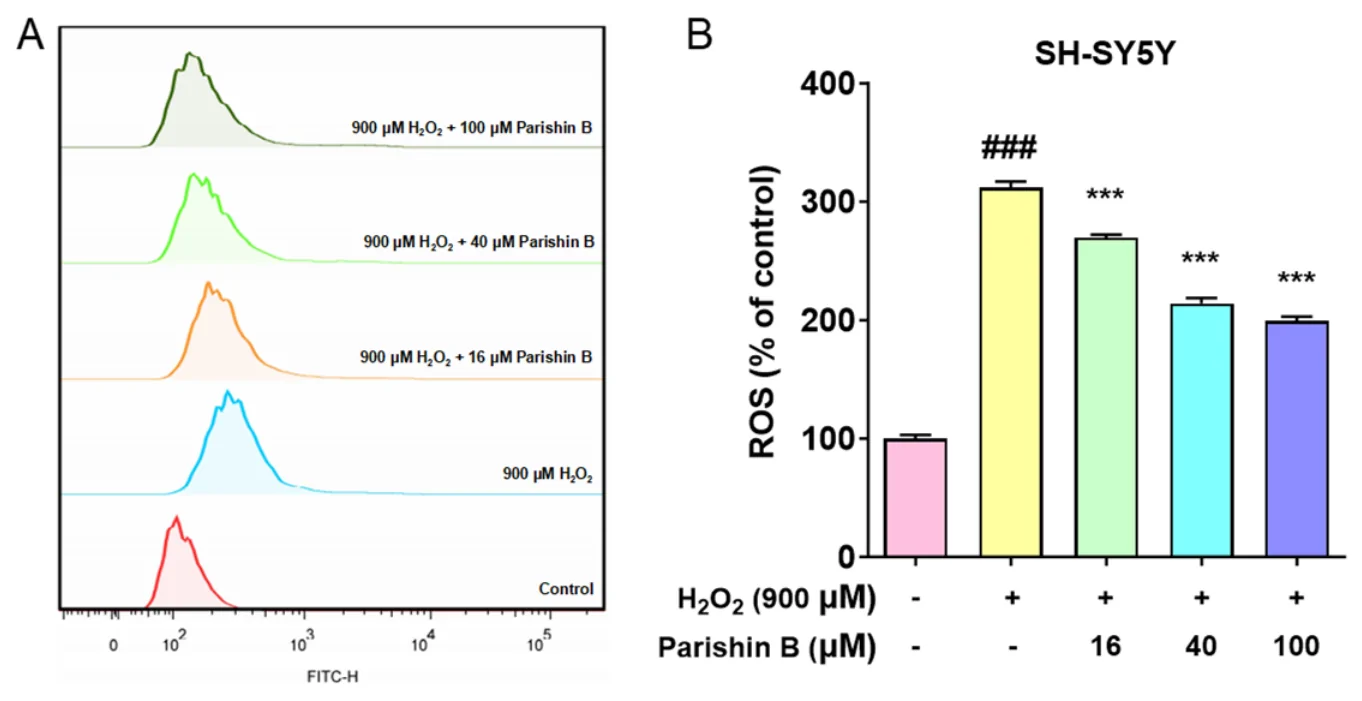
Effect of Parishin B on H_2_O_2_-induced ROS generation in SH-SY5Y cells. (**A**) Representative population peak diagrams of flow cytometry detecting the effect of Parishin B at different concentrations (16, 40, 100 μM) on 900 μM H_2_O_2_-induced ROS generation in SH-SY5Y cells. (**B**) Relative intracellular ROS content of 900 μM H_2_O_2_-induced SH-SY5Y cells pre-treated with Parishin B at different concentrations. Data are expressed as mean ± SD, n = 3, ### *p* < 0.001 vs. control group; *** *p* < 0.001 vs. model group.

ROS. By DCFH-DA staining, intracellular ROS increased significantly after H_2_O_2_ treatment (*p* < 0.001), confirming the model. Pretreatment with 16, 40, and 100 μM Parishin B dose-dependently reduced ROS (Figure 11), with the greatest effect at 100 μM. These results indicated that Parishin B helped maintain redox homeostasis by reducing excessive ROS, which might contribute to its neuroprotective effect.

#### 3.5.3. Anti-Neuroinflammatory Effect of Parishin B

**Figure 12:**
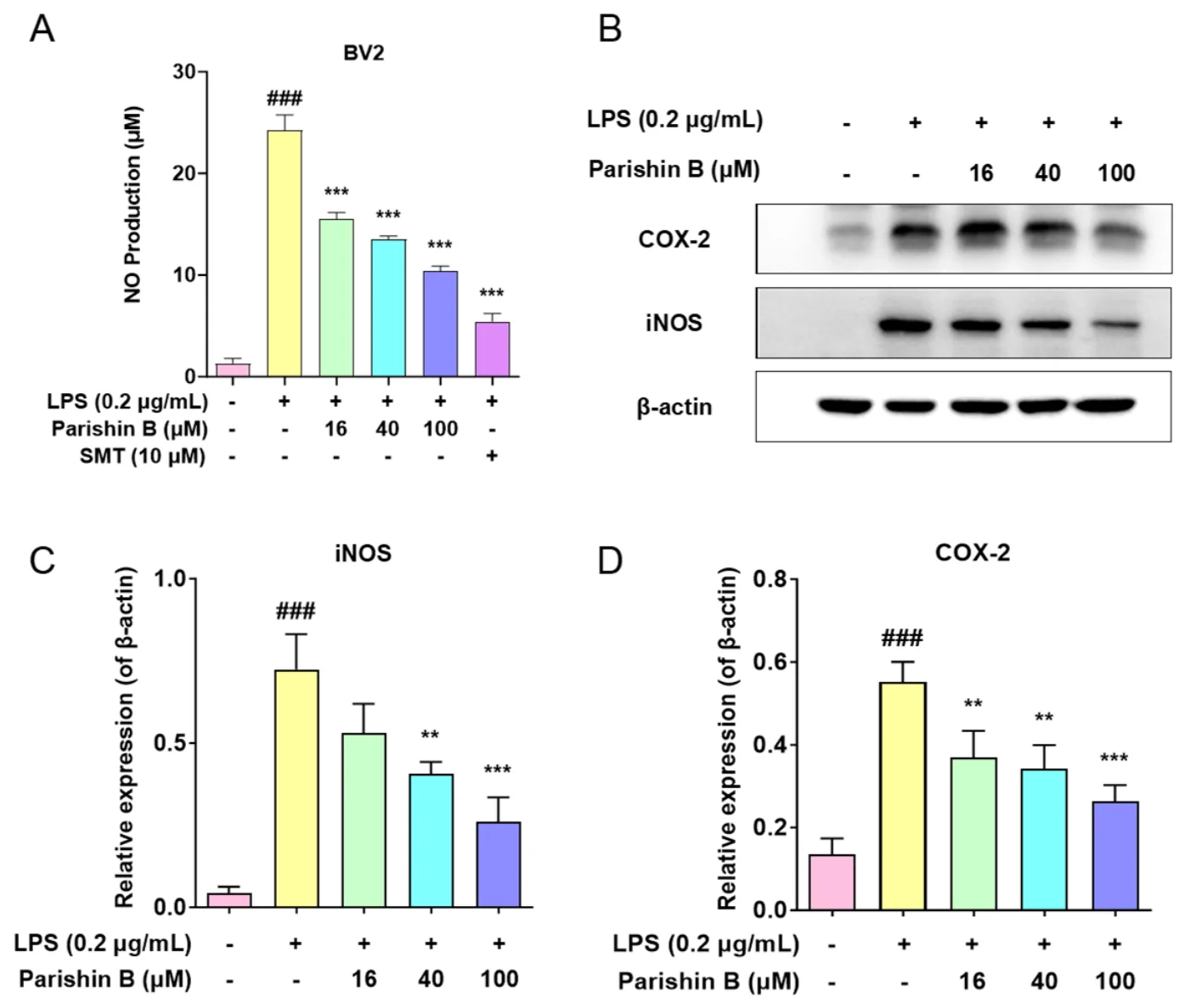
Effects of Parishin B on NO release and iNOS/COX-2 expression in LPS-induced BV-2 microglia. (**A**) Effects of different concentrations of Parishin B on NO release in LPS-induced BV-2 cells. S-methylisothiourea (SMT) was used as a positive control. (**B**) Representative Western blot bands showing the effects of different concentrations of Parishin B on the protein expression of iNOS and COX-2 in LPS-induced BV-2 cells. (**C**,**D**) Relative protein expression of iNOS and COX-2 in LPS-induced BV-2 cells after treatment with different concentrations of Parishin B. Data are presented as mean ± SD, n = 3, ### *p* < 0.001 vs. control group; ** *p* < 0.01, *** *p* < 0.001 vs. model group. Blots from three independent experiments are provided in Figure S2.

NO release. By the Griess assay, 0.2 μg/mL LPS significantly increased NO release from BV-2 cells after 24 h (*p* < 0.001), confirming the neuroinflammation model. Parishin B (16, 40, and 100 μM) inhibited NO release concentration-dependently (Figure 12A). Relative to the LPS model group, the NO inhibition rate was 60.50 ± 1.81% for 100 μM Parishin B and 82.01 ± 2.73% for the SMT positive control (both *p* < 0.001). Thus, Parishin B markedly attenuated LPS-induced neuroinflammation, likely by suppressing microglial activation.

iNOS and COX-2 expression. By Western blot, iNOS and COX-2 were significantly upregulated in the LPS model group versus the control (*p* < 0.001). Co-treatment with 16, 40, and 100 μM Parishin B suppressed this upregulation concentration-dependently (Figure 12B–D), with the strongest effect at 100 μM. These findings, consistent with the NO results, indicated that the anti-inflammatory action of Parishin B involved downregulation of the iNOS/COX-2 pathway.

## 4. Discussion

Using a “rapid screening—efficacy validation—mechanistic exploration” strategy, this study prioritized an anti-AD constituent from *G. elata* water extract with PDE4B as a molecular anchor and characterized its actions. We identified Parishin B as a representative, chemically trackable, developable constituent that directly inhibits PDE4B enzymatic activity, ameliorates behavioral deficits in Al^3+^-induced zebrafish (with no significant difference from donepezil hydrochloride under the tested conditions), and exerts two complementary neuroprotective actions in cultured cells: protection against oxidative injury (apoptosis inhibition and ROS reduction) and anti-neuroinflammation (downregulation of iNOS and COX-2). These findings provide new experimental evidence for the modern development of *G. elata* and for multi-target approaches to AD.

### 4.1. Rationale and Methodological Significance of the Strategy

The “efficacy-first, mechanism-second” strategy used here offers practical advantages. Conventional natural-product studies often follow a linear “in vitro screening → in vivo validation” route, which is prone to “active in vitro, inactive in vivo” false positives. Here, a high-scoring candidate (Parishin B) was rapidly prioritized by docking and then validated directly in whole-animal zebrafish, after which targeted cell experiments were used to explore the mechanism. As a whole-organism model, zebrafish more comprehensively reflect absorption, distribution, and metabolism, and their behavioral readouts correspond directly to core AD-like symptoms [23]. Importantly, because direct affinity-based fishing of binders from a complex herbal matrix is often confounded by co-eluting and high-abundance components, the chemical-profiling-guided, docking-then-validation route adopted here provides a pragmatic alternative for prioritizing developable constituents from natural products.

### 4.2. Binding of Parishin B to PDE4B and Its Role as a Docking Anchor

Docking indicated that Parishin B had the most favorable overall profile among the analyzed constituents (normalized comprehensive score, 0.9333), forming a hydrogen-bond network with key active-site residues (e.g., Gln615, Met519, His234) and interacting with Mg^2+^, a cofactor essential for PDE4B catalysis [48]. The docking score of Parishin B (−9.5 kcal/mol) was numerically favorable relative to that reported for rolipram (approximately −8.5 kcal/mol) [49,50]; however, because AutoDock Vina scores are biased toward large, flexible ligands, these values are used only for relative prioritization rather than as a direct affinity comparison. Critically, the docking prediction was corroborated by a recombinant-enzyme assay showing that Parishin B directly reduces PDE4B catalytic activity (residual activity, 22.1% at 100 μM). PDE4B regulates microglial activation by hydrolyzing cAMP, and its early inhibition improves cognition and reduces neuroinflammation in APP knock-in and 3xTg-AD models [15,51]. Together, these data show that Parishin B can directly inhibit PDE4B, providing a biochemical rationale for the anti-inflammatory effects observed in BV-2 microglia. We note, however, that direct target engagement in cells and in vivo was not established here (Section 4.5).

### 4.3. In Vivo Findings and the Whole-Extract Versus Constituent Relationship

*G. elata* water extract was most effective at 30 μg/mL. Parishin B showed significant effects at 10 μM (≈7.29 μg/mL) and was most effective at 100 μM (≈72.86 μg/mL). Two observations deserve emphasis. First, the whole extract was effective at a mass concentration of 30 μg/mL, and 100 μg/mL GWE produced no additional benefit. This pattern is consistent with the non-linear pharmacokinetics reported for *G. elata* and parishin-type constituents at higher doses [52,53] and with the inverted-U dose–response commonly seen for polyphenol-rich herbal extracts [54,55,56]. More importantly, the fact that the whole extract was active in the AlCl_3_-induced zebrafish model indicates that the anti-AD effect of *G. elata* does not depend on a single constituent but rather reflects a multi-component contribution—a recognized feature of natural products compared with single compounds. Other constituents, such as gastrodin, parishin G, and 4-hydroxybenzyl alcohol, may act through complementary targets (e.g., inhibiting Aβ aggregation, modulating the gut microbiota, or scavenging free radicals) [10,11]. This framing repositions Parishin B not as the sole active principle but as one chemically trackable, developable representative of the herb’s activity. Finally, although the zebrafish model reproduces AD-like behavioral deficits, it cannot fully replicate human AD pathology (e.g., Aβ plaques and tau tangles), and confirmation in mammalian models such as APP/PS1 mice is needed.

### 4.4. Complementary Anti-Neuroinflammatory and Neuroprotective Actions

In vitro, Parishin B acted through two complementary actions that are mechanistically distinct.

In BV-2 microglia, Parishin B concentration-dependently suppressed LPS-induced NO release and downregulated iNOS and COX-2, the core inducible effectors whose overexpression drives excessive NO and prostaglandin E_2_ (PGE_2_) production and amplifies the inflammatory cascade [57,58]. Because our docking and recombinant-enzyme assays identified PDE4B as a molecular anchor of Parishin B, and PDE4B is an established regulator of microglial neuroinflammation through cAMP hydrolysis [13,51], we propose that this anti-inflammatory action may be linked to the PDE4B/cAMP axis: PDE4B inhibition would elevate intracellular cAMP, activate protein kinase A (PKA), and promote cAMP-response element-binding protein (CREB) phosphorylation, which in turn would restrain nuclear factor-κB (NF-κB)-driven transcription of iNOS and COX-2. Interestingly, Parishin B has also been reported to modulate neuroinflammation via the AMPK/SIRT1/NF-κB pathway [8], suggesting convergent regulation through PDE4B-dependent and PDE4B-independent routes. However, since we did not measure these markers, our interpretation focuses on the PDE4B interaction supported by docking and enzymatic data. The detection of Parishin B in rat brain after oral administration of *G. elata* extract [59] supports the plausibility of a central action. We emphasize, however, that none of the intermediate nodes (cAMP, PKA, p-CREB, NF-κB) were measured here; the causal contribution of PDE4B to the observed iNOS/COX-2 downregulation therefore remains a hypothesis to be tested (Section 4.5).

Beyond its action on microglia, Parishin B provided direct neuroprotection in SH-SY5Y cells subjected to oxidative injury, improving viability and attenuating apoptosis, accompanied by a concentration-dependent reduction in intracellular ROS. Oxidative stress is central to AD, as excess ROS damage mitochondria, induce DNA damage, and activate apoptotic cascades [1]. Given the free phenolic hydroxyl group in its metabolite p-hydroxybenzyl alcohol, this neuroprotective effect is most parsimoniously interpreted as a general, target-independent property of the molecule rather than a downstream consequence of PDE4B inhibition. We therefore present it as a complementary neuroprotective action that broadens the benefit of Parishin B—consistent with the well-documented neuroprotective profile of *G. elata* constituents—rather than as a second, PDE4B-dependent pathway.

Taken together, these two actions are complementary rather than synergistic in the strict sense, as no interaction between them was experimentally interrogated. Their combination—restraining microglial inflammation on the one hand and protecting neurons from oxidative injury on the other—mirrors the multi-component, multi-target character often ascribed to *G. elata* and offers a coherent, if still partly hypothetical, framework for the anti-AD potential of Parishin B that warrants the target-engagement validation outlined below.

### 4.5. Limitations and Future Directions

Several limitations should be acknowledged. First, although Parishin B inhibited PDE4B enzymatically in vitro, downstream signaling nodes (cAMP, PKA, p-CREB, and related effectors) were not measured in zebrafish brain tissue, so involvement of the PDE4B–cAMP–PKA–CREB axis remains hypothetical; a cell-based cAMP assay (with PKA inhibition or forskolin as controls) would substantially strengthen this link. Moreover, cellular cAMP levels were not measured in BV-2 cells, and no PDE4B knockdown or other target-dependence experiment was performed; whether the anti-inflammatory effects of Parishin B are mediated through PDE4B therefore remains to be established. In addition, BV-2 cell viability and cell number were not assessed under LPS co-treatment, so a contribution of altered viability or cell number to the NO readout cannot be fully excluded. Second, efficacy was not confirmed in mammalian models, and the zebrafish model cannot fully reproduce all aspects of human AD pathology. Moreover, Aβ and p-tau were not directly measured in this study; the link to AD pathology relies on published model validation [25,26]. In addition, a Parishin B-only group was not included, so the possibility that Parishin B affects baseline activity or color preference independently of AlCl_3_ cannot be fully excluded. Third, the in vivo studies used a post-exposure treatment regimen, whereas the in vitro mechanistic studies used pretreatment in the oxidative injury model and co-treatment in the LPS-induced inflammation model—a widely used paradigm for mechanistic work [60,61]; consequently, the cellular pathways identified in vitro may not fully account for the in vivo effects, and direct evidence of pathway engagement in brain tissue is currently lacking. Fourth, because parishins are citric acid esters of gastrodin and can hydrolyze in vivo, whether the observed effects are mediated by intact Parishin B or by its metabolite gastrodin remains to be clarified through metabolic and exposure studies. Fifth, the enzymatic inhibition required relatively high concentrations of Parishin B, and its brain exposure after oral dosing may not reach these levels; quantitative IC_50_ determination and pharmacokinetic characterization are therefore warranted. In addition, only three concentrations (1, 10, and 100 μM) were tested in the PDE4B inhibition assay, so an IC_50_ could not be determined, and docking was performed with human PDE4B structures, whereas the enzymatic assay used rat PDE4B; thus, the two are not from the same species. Future studies in APP/PS1 transgenic mice should evaluate behavioral and pathological outcomes and systematically examine key signaling molecules (cAMP, p-PKA, p-CREB, iNOS, and COX-2) in brain tissue, thereby providing direct in vivo evidence for the proposed PDE4B/cAMP axis.

## 5. Conclusions

Focusing on PDE4B as a molecular anchor within the AD-related neuroinflammatory pathway, this study integrated chemical profiling, computational prioritization, enzymatic validation, and phenotypic assessment to identify a developable anti-AD constituent of *G. elata* water extract. Parishin B directly inhibited PDE4B enzymatic activity and, in cultured cells, exerted two complementary neuroprotective actions—protection against oxidative injury (apoptosis inhibition and ROS reduction) and anti-neuroinflammation (downregulation of iNOS and COX-2). In the Al^3+^-induced zebrafish model, Parishin B ameliorated behavioral deficits, showing no significant difference from donepezil hydrochloride under the present conditions. At the whole-extract level, *G. elata* water extract likewise ameliorated behavioral deficits, providing pharmacological support for its traditional use against forgetfulness. Collectively, Parishin B is a developable lead worthy of further target-engagement validation, and the integrated strategy used here may serve as a reference for prioritizing active constituents from other traditional Chinese medicines.

## Figures and Tables

**Table 1:** Major constituents identified in *G. elata* water extract.

Peak No.	Identification	tR (min)	Formula	[M − H]^−^ (*m*/*z*)	MS^2^ Fragment Ions (*m*/*z*)	Mass Error (ppm)	Ref.
1	Lactobionic acid	0.97	C_12_H_22_O_12_	357.1059	75.0095; 129.0200	8.814	Putatively identified
2	Sucrose	1.01	C_12_H_22_O_11_	341.1109	179.0572; 89.0249	9.065	[41]
3	Isomaltose	1.01	C_12_H_22_O_11_	341.1109	89.0249; 59.0146; 71.0146	9.065	Putatively identified
4	Gastrodin	4.82	C_13_H_18_O_7_	285.0995	123.0458	9.192	[41]
5	Magnolol	10.19	C_18_H_18_O_2_	265.1215	127.0522	−2.966	[42]
6	Gastrodin A	10.35	C_19_H_28_O_12_	447.1530	323.1002	7.374	[43]
7	Parishin E/G	13.94	C_19_H_24_O_13_	459.1169	111.0095; 87.0093; 173.0101; 129.0200	7.826	[43]
8	Parishin E/G	14.13	C_19_H_24_O_13_	459.1169	111.0095; 87.0093; 173.0100; 57.0353	7.760	[43]
9	S-(4-hydroxybenzyl)-glutathione	14.41	C_17_H_23_N_3_O_7_S	412.1209	306.0786	8.694	[43]
10	Parishin I/V	18.32	C_38_H_50_O_24_	889.2689	780.1254; 585.1484; 423.0951; 111.0093	9.032	[43]
11	Parishin B/C	18.35	C_32_H_40_O_19_	727.2141	423.0955; 161.0463; 111.0094; 397.1158	8.395	[44]
12	Parishin I/V	18.62	C_38_H_50_O_24_	889.2679	585.1496; 323.1002; 111.0093; 423.0967	7.997	[43]
13	Parishin H/M	18.90	C_33_H_42_O_20_	757.2236	453.1061; 423.0953; 161.0462; 111.0094	6.603	[43]
14	Parishin B/C	19.26	C_32_H_40_O_19_	727.2134	423.0953; 161.0462; 111.0094; 397.1158	7.391	[44]
15	Parishin F	21.80	C_51_H_66_O_30_	1157.3601	889.2679; 727.2146	3.969	[43]
16	Parishin A	21.80	C_45_H_56_O_25_	995.2976	727.2142; 423.0957; 397.1160; 161.0460	−5.107	[43]

Slash-labeled assignments (Parishin B/C, E/G, I/V, and H/M) are tentative, based on accurate mass and MS^2^ fragments; the corresponding isomers could not be discriminated by LC-MS, and confirmation requires authentic standards.

**Table 2:** Docking energies and hydrogen-bonding residues of the 14 selected constituents with PDE4B (4MYQ/1XMY).

No.	Compound Name	PDE4B (PDB ID: 4MYQ)		PDE4B (PDB ID: 1XMY)	
		Affinity (kcal/mol)	Hydrogen-Bonded Residues and Metal-Ion Contacts	Affinity (kcal/mol)	Hydrogen-Bonded Residues and Metal-Ion Contacts
1	Gastrodin	−7.3	HIS-406, TYR-405, SER-614	−7.9	ASN-395, ASP-392, MET-347, THR-345, HIS-234, HIS-278, Mg^2+^, Zn^2+^
2	Magnolol	−8.7	No hydrogen bond	−8.9	GLN-443
3	Gastrodin A	−8.4	ASP-564, HIS-450, ASN-455, GLN-456	−8.9	ASN-283, MET-347, ASP-275, ASP-392, HIS-234, Mg^2+^
4	Parishin E	−9.2	HIS-450, GLU-476, TRP-578, Mg^2+^	−8.1	GLN-443, HIS-234, ASP-275, HIS-278, Mg^2+^, Zn^2+^
5	Parishin G	−9.4	GLN-615, ASN-567, ASN-455, ASP-518, Mg^2+^	−8.4	ASP-275, HIS-234, GLN-443, Mg^2+^, Zn^2+^
6	S-(4-hydroxybenzyl)-glutathione	−8.7	GLN-615, MET-519, HIS-406, HIS-450, Mg^2+^	−7.4	GLN-443, HIS-234, ASP-392, HIS-278, Mg^2+^, Zn^2+^
7	Parishin I	−7.0	HIS-406, ASN-455, GLN-589, LYS-677, GLN-615, Mg^2+^, Zn^2+^	−7.5	HIS-238, GLU-304, HIS-234, GLN-417, Mg^2+^, Zn^2+^
8	Parishin V	−8.1	ASP-564, MET-519, GLN-615, LYS-677, ASN-455, GLN-456, Mg^2+^	−7.7	MET-347, ASP-392, HIS-234, GLN-443, CYS-432, MET-431
9	Parishin B	−9.5	GLN-615, CYS-671, LEU-674, MET-519, ASP-518, GLU-476, HIS-450, Mg^2+^	−8.7	GLN-417, GLN-443, SER-282, THR-345, HIS-234, HIS-278, Mg^2+^
10	Parishin C	−8.3	SER-520, MET-519, ASP-447, GLN-615, HIS-406	−7.7	GLN-443, ASN-395, HIS-234, THR-345, MET-347, GLU-304
11	Parishin H	−8.2	ASN-455, ASP-518, HIS-406, Mg^2+^	−7.6	TYR-233, HIS-234, ASP-392, HIS-278, GLU-304, Zn^2+^
12	Parishin M	−8.1	SER-614, GLN-615, HIS-406, ASP-518, ASN-455	−8.2	ASN-395, HIS-234, MET-347, GLU-304, Mg^2+^, Zn^2+^
13	Parishin F	−4.1	ASN-455, HIS-450, HIS-406, LYS-677, SER-520, MET-519, SER-614, GLN-615, ASP-564, Mg^2+^	−6.0	HIS-234, ASP-275, GLU-304, ASN-283, MET-347, Mg^2+^
14	Parishin A	−8.4	ASN-455, LYS-677, GLY-673, GLN-615	−7.5	HIS-278, ASN-283, HIS-234, ASN-395, GLN-443, SER-442, Mg^2+^

## Data Availability

The original contributions presented in this study are included in the article/Supplementary Materials. Further inquiries can be directed to the corresponding authors.

## Supplementary Materials

The following supporting information can be downloaded at: https://www.mdpi.com/article/10.3390/biology15201830/s1, Figure S1: Molecular docking visualization of the 14 selected components from *Gastrodia elata* with PDE4B (PDB IDs: 4MYQ and 1XMY); Figure S2: Original Western blot images for Figure 12. Western blot images from three independent experiments for iNOS, COX-2, and β-actin in LPS-induced BV-2 cells. Table S1: Normalized comprehensive docking scores of the 13 characteristic components from *Gastrodia elata* with PDE4B; Table S2: Raw fluorescence polarization (mP) values and calculated % of Activity for PDE4B enzymatic inhibition assay; Table S3: Behavioral parameters of zebrafish larvae in the AlCl_3_-induced AD model treated with *Gastrodia elata* water extract (GWE; corresponding to Figure 5); Table S4: Raw tracking data of zebrafish larvae in the AlCl_3_-induced AD model treated with *Gastrodia elata* water extract (GWE); Table S5: Behavioral parameters of zebrafish larvae in the AlCl_3_-induced AD model treated with Parishin B (corresponding to Figure 7); Table S6: Raw tracking data of zebrafish larvae in the AlCl_3_-induced AD model treated with Parishin B.

## Abbreviations

The following abbreviations are used in this manuscript:

AD	Alzheimer’s disease
Aβ	β-amyloid
cAMP	cyclic adenosine monophosphate
COX-2	cyclooxygenase-2
DPZ	donepezil hydrochloride
EGCG	epigallocatechin gallate
GWE	*Gastrodia elata* Blume water extract
HESI	heated electrospray ionization source
hpf	hours post-fertilization
iNOS	inducible nitric oxide synthase
LPS	lipopolysaccharide
MTT	methylthiazolyldiphenyl-tetrazolium bromide
NF-κB	nuclear factor-κB
NO	nitric oxide
PBS	phosphate-buffered saline
PDE4B	phosphodiesterase 4B
PGE_2_	prostaglandin E_2_
PI	propidium iodide
PKA	protein kinase A
PMSF	phenylmethylsulfonyl fluoride
ROS	reactive oxygen species
SDS-PAGE	sodium dodecyl sulfate-polyacrylamide gel electrophoresis
SMT	S-methylisothiourea
UHPLC	ultra-high-performance liquid chromatography
UPLC-ESI-Q-Orbitrap MS	ultra-performance liquid chromatograph coupled with electrospray ionization quadrupole Orbitrap mass spectrometry
