# Supplementary material for: A Docking-Guided Strategy Prioritizes Parishin B as a Potential Anti-Alzheimer’s Constituent of Gastrodia elata Blume
Source: Biology (Basel). 2026 Oct 13;15(20):1830. doi: 10.3390/biology15201830 (PMC13701641; doi:10.3390/biology15201830)
Supplement: Supplementary file 1 [file biology-15-01830-s001.zip › biology-4577277-supplementary.pdf]

Supplementary Materials

A Docking-Guided Strategy Prioritizes Parishin B as a Potential Anti-Alzheimer’s Constituent of *Gastrodia elata* Blume

Contents

1. **Figure S1.** Molecular docking visualization of the 14 selected components from *Gastrodia elata* with PDE4B (PDB IDs: 4MYQ and 1XMY).
2. **Figure S2.** Original Western blot images for Figure 12. Western blot images from three independent experiments for iNOS, COX-2, and β-actin in LPS-induced BV-2 cells.
3. Procedure for calculating normalized comprehensive binding energies and corresponding scoring table.
4. **Table S2.** Raw fluorescence polarization (mP) values and calculated % of Activity for PDE4B enzymatic inhibition assay.
5. **Table S3.** Behavioral parameters of zebrafish larvae in the $AlCl_3$-induced AD model treated with *Gastrodia elata* water extract (GWE; corresponding to Figure 5).
6. **Table S4.** Raw tracking data of zebrafish larvae in the $AlCl_3$-induced AD model treated with *Gastrodia elata* water extract (GWE).
7. **Table S5.** Behavioral parameters of zebrafish larvae in the $AlCl_3$-induced AD model treated with Parishin B (corresponding to Figure 7).
8. **Table S6.** Raw tracking data of zebrafish larvae in the $AlCl_3$-induced AD model treated with Parishin B.

A

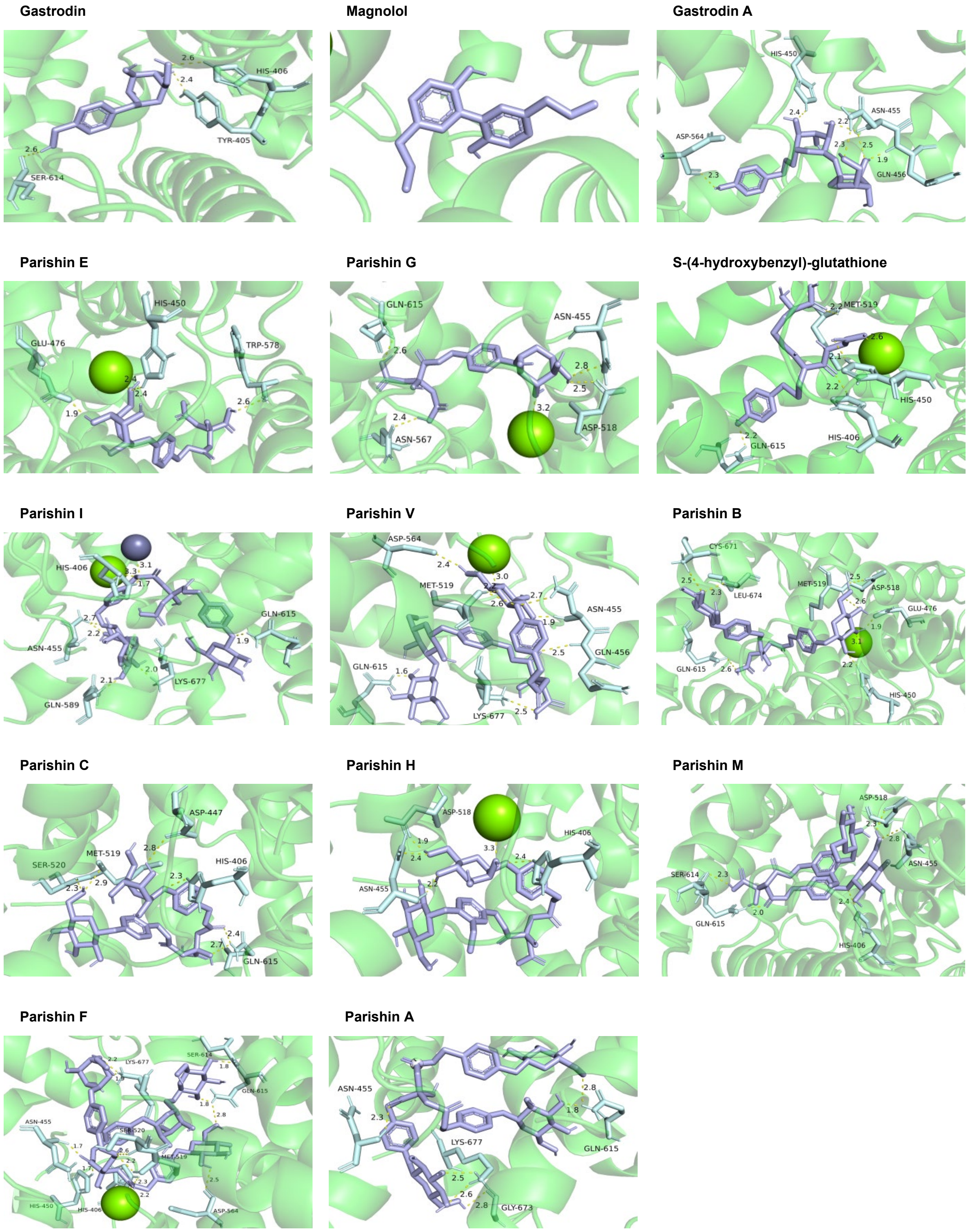
Gastrodin
HIS-406
TYR-405
SER-614
Magnolol
Gastrodin A
HIS-450
ASN-455
ASP-564
GLN-456
Parishin E
HIS-450
GLU-476
TRP-578
Parishin G
GLN-615
ASN-455
ASN-567
ASP-518
S-(4-hydroxybenzyl)-glutathione
MET-519
HIS-450
HIS-406
GLN-615
Parishin I
HIS-406
GLN-615
ASN-455
LYS-677
GLN-589
Parishin V
ASP-564
MET-519
ASN-455
GLN-456
GLN-615
LYS-677
Parishin B
CYS-671
LEU-674
MET-519
ASP-518
GLU-476
GLN-615
HIS-450
Parishin C
ASP-447
MET-519
SER-520
HIS-406
GLN-615
Parishin H
ASP-518
HIS-406
ASN-455
Parishin M
ASP-518
ASN-455
SER-614
GLN-615
HIS-406
Parishin F
Parishin A
ASN-455
LYS-677
GLN-615
GLY-673

B

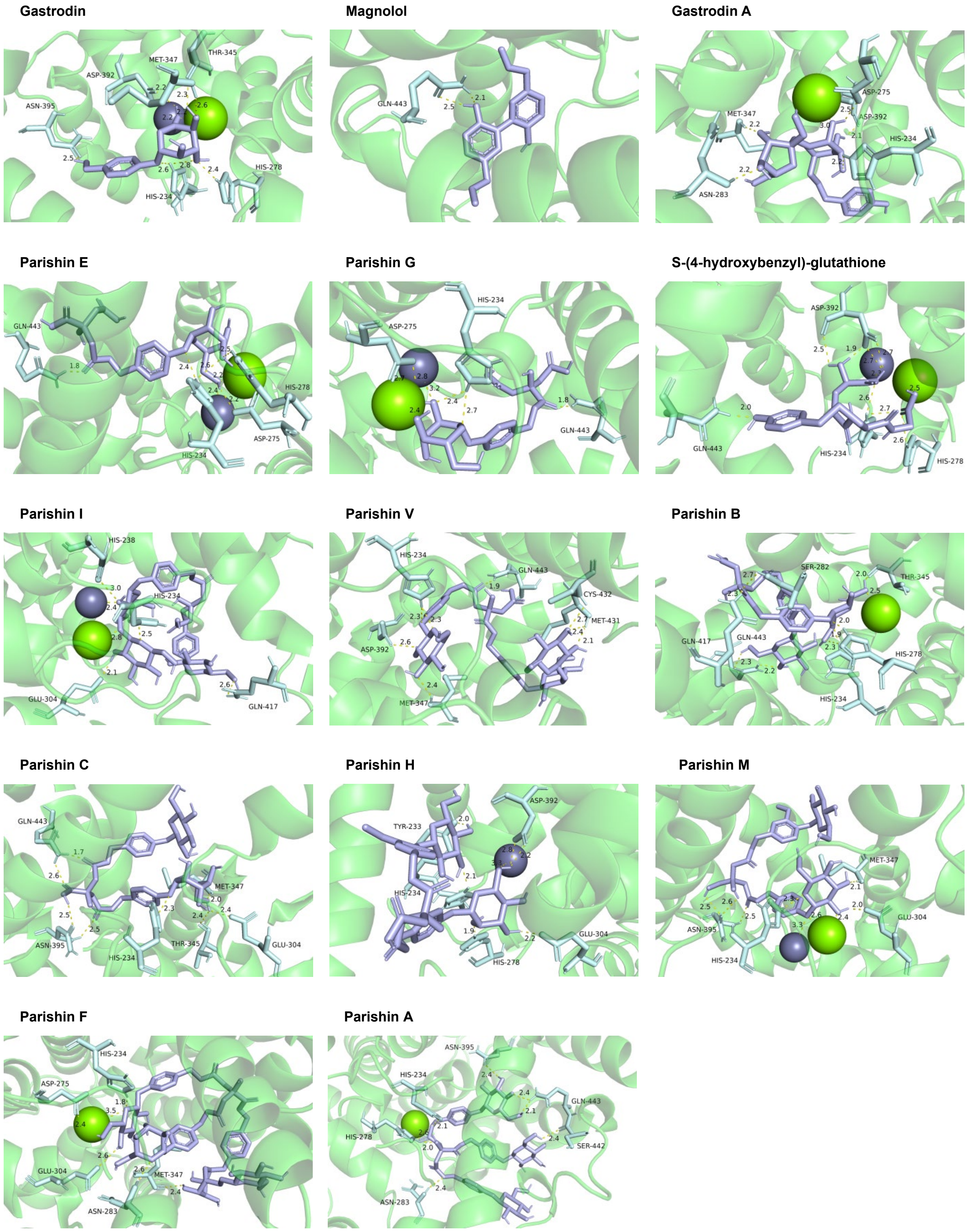

Figure S1. Molecular docking visualization of the 14 selected components from *Gastrodia elata* with PDE4B (PDB IDs: 4MYQ and 1XMY). (A) Docking poses with 4MYQ. (B) Docking poses with 1XMY.

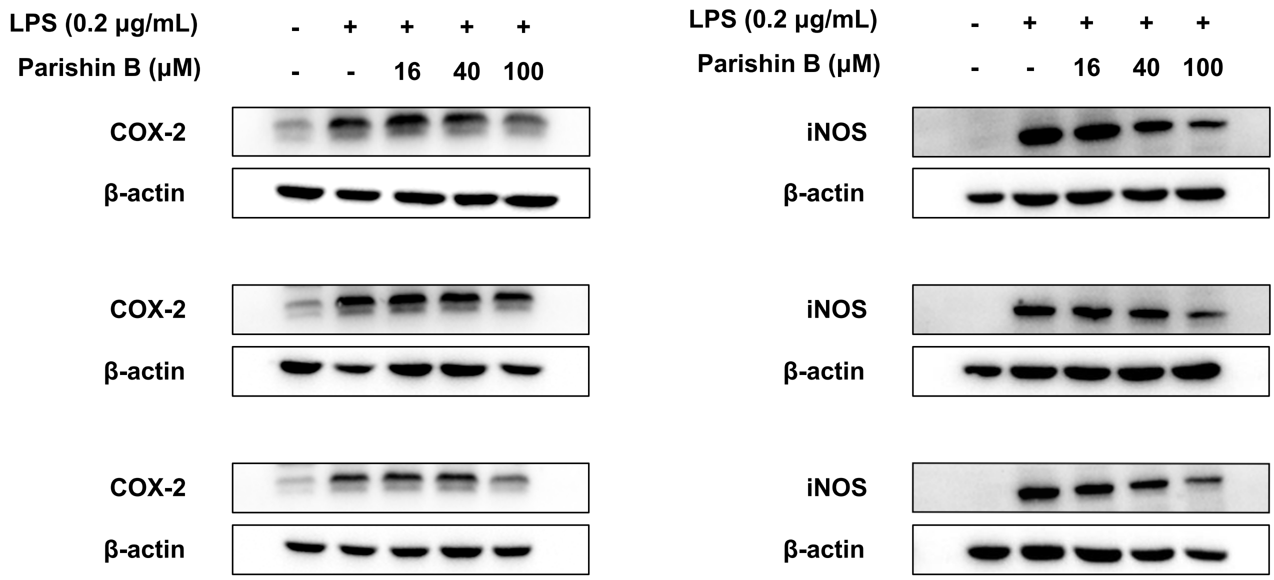

Figure S2. Original Western blot images for Figure 12. Western blot images from three independent experiments for iNOS, COX-2, and β-actin in LPS-induced BV-2 cells. COX-2 dark-field and bright-field images were captured separately and are shown without merging.

3. Procedure for calculating normalized comprehensive binding energies and corresponding scoring table.

This section employs a normalized comprehensive binding energy method to quantitatively evaluate the docking results of 13 characteristic components from *Gastrodia elata* (after excluding Parishin F, which showed no effective binding) with two crystal structures of PDE4B (4MYQ and 1XMY), aiming to identify the candidate component with the optimal overall binding affinity.

(1) Calculation Methods and Formulas

The two crystal structures were assigned equal weights: $w_1 = w_2 = 0.5$.

Since binding energy is a smaller-the-better indicator, the normalization formula is:

$E_n = (E_{max} - E)/ (E_{max} - E_{min})$

The comprehensive score formula is:

$E_{comprehensive} = w_1 \times E_{1n} + w_2 \times E_{2n}$

Where: E_n represents the normalized binding energy; E_{max} and E_{min} represent the maximum and minimum binding energies for a given target; E_{1n} and E_{2n} represent the normalized binding energies for 4MYQ and 1XMY, respectively.

(2) Key Parameters

For the PDE4B target, the binding energy ranges of the two crystal structures were as follows. For 4MYQ, the maximum binding energy was −7.0 kcal/mol (Parishin I) and the minimum was −9.5 kcal/mol (Parishin B), giving a range of **2.5** kcal/mol. For 1XMY, the maximum binding energy was −7.4 kcal/mol (S-(4-hydroxybenzyl)-glutathione) and the minimum was −8.9 kcal/mol (Magnolol and Gastrodin A), giving a range of **1.5** kcal/mol. The extreme values for each target were used as the basis for subsequent normalization calculations.

(3) Normalization Calculation and Comprehensive Scores

Based on the extreme values for each target and the calculation formulas described above, the binding energies of the 13 characteristic components from *Gastrodia elata* were normalized for the 4MYQ and 1XMY targets. Using the weight coefficients ($w_1 = 0.5$, $w_2 = 0.5$), the normalized comprehensive binding energy scores for each component were derived. All calculated results are presented with four decimal places. The original binding energies, normalized values, and comprehensive scores are shown in Table S1.

Table S1.

Normalized comprehensive docking scores of the 13 characteristic components from *Gastrodia elata* with PDE4B.

No.	Compound	4MYQ E (kcal/mol)	1XMY E (kcal/mol)	4MYQ E_n	1XMY E_n	$E_{comprehensive}$	Rank
1	Gastrodin	−7.3	−7.9	0.1200	0.3333	0.2267	12
2	Magnolol	−8.7	−8.9	0.6800	1.0000	0.8400	2
3	Gastrodin A	−8.4	−8.9	0.5600	1.0000	0.7800	4
4	Parishin E	−9.2	−8.1	0.8800	0.4667	0.6733	5
5	Parishin G	−9.4	−8.4	0.9600	0.6667	0.8133	3
6	S-(4-hydroxybenzyl)-glutathione	−8.7	−7.4	0.6800	0.0000	0.3400	8
7	Parishin I	−7.0	−7.5	0.0000	0.0667	0.0333	13
8	Parishin V	−8.1	−7.7	0.4400	0.2000	0.3200	9
9	Parishin B	−9.5	−8.7	1.0000	0.8667	0.9333	1
10	Parishin C	−8.3	−7.7	0.5200	0.2000	0.3600	7
11	Parishin H	−8.2	−7.6	0.4800	0.1333	0.3067	11
12	Parishin M	−8.1	−8.2	0.4400	0.5333	0.4867	6
13	Parishin A	−8.4	−7.5	0.5600	0.0667	0.3133	10

(4) Analytical Conclusion

Parishin B achieved the highest normalized comprehensive score (0.9333), demonstrating strong and stable binding affinity to both PDE4B crystal structures. Its overall binding characteristics are significantly superior to those of the other components, establishing it as the core candidate compound for subsequent functional validation.

Table S2.

Raw fluorescence polarization (mP) values and calculated % of Activity for PDE4B enzymatic inhibition assay.

Group	mP_1	mP_2	mP_3	% Activity (Mean ± SD)
Reference Control	109	101	106	—
Positive Control	237	201	200	100.0 ± 19.6
Roflumilast (0.1 nM)	159	151	145	43.2 ± 6.5
Parishin B (1 μM)	199	177	189	77.3 ± 10.3
Parishin B (10 μM)	162	177	164	58.1 ± 7.6
Parishin B (100 μM)	131	138	118	22.1 ± 9.5

The percentage of residual activity was calculated as: % of Activity = $\frac{\text{(mP value from Test Inhibitor - mP value from Reference Control)}}{\text{(mP value from Positive Control - mP value from Reference Control)}} \times 100$, Where mP_{Ref} = 105.3 and mP_{Pos} = 212.7 are the mean values of the Reference and Positive Controls, respectively. Data represent three independent measurements (*n* = 3).

Table S3.

Behavioral parameters of zebrafish larvae in the $AlCl_3$-induced AD model treated with *Gastrodia elata* water extract (GWE; corresponding to Figure 5)

Group	Total swimming distance (mm) (mean ± SD)	*P* vs. Control	*P* vs. model	Swimming speed (mm/s) (mean ± SD)	*P* vs. Control	*P* vs. model	Cumulative residence time in the blue zone (s) (mean ± SD)	*P* vs. Control	*P* vs. model
Control	2326.393 ± 201.019	—	< 0.001	3.893 ± 0.325	—	< 0.001	125.493 ± 3.128	—	< 0.001
$AlCl_3$ model	1372.500 ± 136.165	< 0.001	—	2.291 ± 0.226	< 0.001	—	74.513 ± 7.652	< 0.001	—
$AlCl_3$ + GWE 10 μg/mL	1681.718 ± 97.654	0.002	0.017	2.807 ± 0.163	0.001	0.017	102.227 ± 8.747	< 0.001	< 0.001
$AlCl_3$ + GWE 30 μg/mL	1825.713 ± 50.100	0.013	0.002	3.052 ± 0.084	0.011	0.002	118.893 ± 8.428	0.232	< 0.001
$AlCl_3$ + GWE 100 μg/mL	1660.747 ± 73.574	0.002	0.023	2.773 ± 0.124	0.002	0.022	119.100 ± 12.638	0.247	< 0.001
$AlCl_3$ + Donepezil 10 μM	1877.993 ± 51.484	0.021	0.001	3.143 ± 0.088	0.018	0.001	114.840 ± 12.341	0.058	< 0.001

n = 6 per group. *P* values were calculated using one-way ANOVA followed by LSD or Dunnett's T3 post-hoc test. Dunnett's T3 was applied when equal variances were not assumed, as determined by Levene's test.

Table S4.

Raw tracking data of zebrafish larvae in the $AlCl_3$-induced AD model treated with *Gastrodia elata* water extract (GWE)

Trial	Well	Group	Total swimming distance (mm)	Swimming speed (mm/s)	Cumulative residence time in the blue zone (s)
1	A1	Control	2134.82	3.60077	121.4
1	A2	Control	2422.41	4.04301	125.04
1	A3	Control	2290.63	3.8246	124.88
1	B1	Control	2673.35	4.46005	123.36
1	B2	Control	2143.69	3.58982	128.68
1	B3	Control	2293.46	3.83676	129.6
2	A1	$AlCl_3$ model	1520.49	2.53601	82.96
2	A2	$AlCl_3$ model	1473.13	2.45833	82.44
2	A3	$AlCl_3$ model	1160.76	1.93822	65.68
2	B1	$AlCl_3$ model	1264.34	2.11541	74.04
2	B2	$AlCl_3$ model	1436.46	2.39713	65.72
2	B3	$AlCl_3$ model	1379.82	2.3017	76.24
3	A1	$AlCl_3$ + Donepezil 10 μM	1925.3	3.21333	126.28
3	A2	$AlCl_3$ + Donepezil 10 μM	1907.22	3.18316	128.2
3	A3	$AlCl_3$ + Donepezil 10 μM	1821.66	3.05462	105.24
3	B1	$AlCl_3$ + Donepezil 10 μM	1805.53	3.00929	96.72
3	B2	$AlCl_3$ + Donepezil 10 μM	1917.74	3.19922	120
3	B3	$AlCl_3$ + Donepezil 10 μM	1890.51	3.19753	112.6
4	A1	$AlCl_3$ + GWE 10 μg/mL	1600.23	2.6724	95.12
4	A2	$AlCl_3$ + GWE 10 μg/mL	1816.18	3.03141	112.88
4	A3	$AlCl_3$ + GWE 10 μg/mL	1602.87	2.67502	93.12
4	B1	$AlCl_3$ + GWE 10 μg/mL	1706.61	2.84643	109.96
4	B2	$AlCl_3$ + GWE 10 μg/mL	1592.42	2.65598	95.08
4	B3	$AlCl_3$ + GWE 10 μg/mL	1772	2.95747	107.2
5	A1	$AlCl_3$ + GWE 30 μg/mL	1877.57	3.1343	123.96
5	A2	$AlCl_3$ + GWE 30 μg/mL	1795.53	2.99575	108.84
5	A3	$AlCl_3$ + GWE 30 μg/mL	1782.69	2.97532	122.44
5	B1	$AlCl_3$ + GWE 30 μg/mL	1900.61	3.17827	127.16
5	B2	$AlCl_3$ + GWE 30 μg/mL	1804.23	3.02744	123.36
5	B3	$AlCl_3$ + GWE 30 μg/mL	1793.65	2.99781	107.6
6	A1	$AlCl_3$ + GWE 100 μg/mL	1727.7	2.88875	123
6	A2	$AlCl_3$ + GWE 100 μg/mL	1530.9	2.55337	116.88
6	A3	$AlCl_3$ + GWE 100 μg/mL	1707.36	2.84845	121.44
6	B1	$AlCl_3$ + GWE 100 μg/mL	1618.34	2.70373	97.8
6	B2	$AlCl_3$ + GWE 100 μg/mL	1693.95	2.82533	136.96
6	B3	$AlCl_3$ + GWE 100 μg/mL	1686.23	2.81734	118.52

All parameters were obtained from center-point tracking.

Table S5.

Behavioral parameters of zebrafish larvae in the $AlCl_3$-induced AD model treated with Parishin B (corresponding to Figure 7).

Group	Total swimming distance (mm) (mean ± SD)	P vs. Control	P vs. model	Swimming speed (mm/s) (mean ± SD)	P vs. Control	P vs. model	Cumulative residence time in the blue zone (s) (mean ± SD)	P vs. Control	P vs. model
Control	2071.693 ± 173.159	—	< 0.001	3.457 ± 0.289	—	< 0.001	138.807 ± 9.695	—	< 0.001
$AlCl_3$ model	848.934 ± 87.399	< 0.001	—	1.416 ± 0.146	< 0.001	—	71.047 ± 9.398	< 0.001	—
$AlCl_3$ + Parishin B 10 μM	1488.820 ± 91.430	0.001	< 0.001	2.487 ± 0.154	0.001	< 0.001	111.547 ± 11.878	< 0.001	< 0.001
$AlCl_3$ + Parishin B 30 μM	1644.648 ± 213.470	0.043	0.001	2.749 ± 0.354	0.044	0.001	123.213 ± 9.982	0.029	< 0.001
$AlCl_3$ + Parishin B 100 μM	1847.053 ± 277.503	0.743	0.002	3.089 ± 0.467	0.768	0.002	122.353 ± 16.388	0.022	< 0.001
$AlCl_3$ + Donepezil 10 μM	1919.112 ± 214.411	0.907	< 0.001	3.213 ± 0.347	0.919	< 0.001	132.120 ± 11.918	0.334	< 0.001

n = 6 per group. *P* values were calculated using one-way ANOVA followed by LSD or Dunnett's T3 post-hoc test. Dunnett's T3 was applied when equal variances were not assumed, as determined by Levene's test.

Table S6.

Raw tracking data of zebrafish larvae in the $AlCl_3$-induced AD model treated with Parishin B.

Trial	Well	Group	Total swimming distance (mm)	Swimming speed (mm/s)	Cumulative residence time in the blue zone (s)
1	A1	Control	2175.33	3.63111	145.84
1	A2	Control	1843.9	3.07644	129.2
1	A3	Control	2235.93	3.73103	147.6
1	B1	Control	1912.84	3.19126	128
1	B2	Control	2012.7	3.35943	133.24
1	B3	Control	2249.46	3.75335	148.96
2	A1	$AlCl_3$ model	730.103	1.21822	75.72
2	A2	$AlCl_3$ model	947.562	1.58106	68.88
2	A3	$AlCl_3$ model	910.916	1.52022	87.96
2	B1	$AlCl_3$ model	870.122	1.45146	63.68
2	B2	$AlCl_3$ model	753.923	1.25796	66.04
2	B3	$AlCl_3$ model	880.975	1.46937	64
3	A1	$AlCl_3$ + Donepezil 10 μM	2129.12	3.55185	149.92
3	A2	$AlCl_3$ + Donepezil 10 μM	1942.02	3.23994	140.28
3	A3	$AlCl_3$ + Donepezil 10 μM	1574.04	2.6704	119.28
3	B1	$AlCl_3$ + Donepezil 10 μM	1761.37	2.93856	119.4
3	B2	$AlCl_3$ + Donepezil 10 μM	2103.32	3.52102	131.8
3	B3	$AlCl_3$ + Donepezil 10 μM	2004.8	3.35632	132.04
4	A1	$AlCl_3$ + Parishin B 10 μM	1413.32	2.35869	111.76
4	A2	$AlCl_3$ + Parishin B 10 μM	1540.34	2.5741	126.04
4	A3	$AlCl_3$ + Parishin B 10 μM	1430.93	2.38966	107.48
4	B1	$AlCl_3$ + Parishin B 10 μM	1619.66	2.70847	124.44
4	B2	$AlCl_3$ + Parishin B 10 μM	1387.61	2.31778	95.56
4	B3	$AlCl_3$ + Parishin B 10 μM	1541.06	2.57289	104
5	A1	$AlCl_3$ + Parishin B 30 μM	1978.53	3.30129	131.8
5	A2	$AlCl_3$ + Parishin B 30 μM	1530.2	2.55408	124.88
5	A3	$AlCl_3$ + Parishin B 30 μM	1573.34	2.63505	121.52
5	B1	$AlCl_3$ + Parishin B 30 μM	1512.21	2.52642	127.28
5	B2	$AlCl_3$ + Parishin B 30 μM	1436.15	2.40707	104.2
5	B3	$AlCl_3$ + Parishin B 30 μM	1837.46	3.06919	129.6
6	A1	$AlCl_3$ + Parishin B 100 μM	1644.55	2.74348	103.96
6	A2	$AlCl_3$ + Parishin B 100 μM	1642.54	2.74838	100.32
6	A3	$AlCl_3$ + Parishin B 100 μM	2138.02	3.56812	138.52
6	B1	$AlCl_3$ + Parishin B 100 μM	1857	3.10909	133.76
6	B2	$AlCl_3$ + Parishin B 100 μM	2225.54	3.73563	133.52
6	B3	$AlCl_3$ + Parishin B 100 μM	1574.67	2.63181	124.04

All parameters were obtained from center-point tracking.
